# Recent urbanization in China is correlated with a Westernized microbiome encoding increased virulence and antibiotic resistance genes

**DOI:** 10.1186/s40168-017-0338-7

**Published:** 2017-09-15

**Authors:** Kathryn Winglee, Annie Green Howard, Wei Sha, Raad Z. Gharaibeh, Jiawu Liu, Donghui Jin, Anthony A. Fodor, Penny Gordon-Larsen

**Affiliations:** 1 Department of Bioinformatics and Genomics, University of North Carolina at Charlotte, Charlotte, NC 28223 USA; 2 Department of Biostatistics, Gillings School of Global Public Health, University of North Carolina at Chapel Hill, Chapel Hill, NC 27516 USA; 3 Department of Bioinformatics and Genomics, University of North Carolina at Charlotte, Kannapolis, NC 28081 USA; 4 Bioinformatics Services Division, Department of Bioinformatics and Genomics, University of North Carolina at Charlotte, Charlotte, NC 28081 USA; 5 Department of Nutrition and Chronic Disease Prevention, Hunan Center for Disease Control and Prevention, Changsha, Hunan Province 410005 China; 6 Department of Nutrition, Gillings School of Global Public Health, University of North Carolina, Chapel Hill, NC 27516 USA; 7 Department of Medicine, Division of Gastroenterology, University of Florida, CGRC, Gainesville, FL 32610 USA

**Keywords:** Microbiome, Urbanization, China, Metagenomics, Metabolome

## Abstract

**Background:**

Urbanization is associated with an increased risk for a number of diseases, including obesity, diabetes, and cancer, which all also show associations with the microbiome. While microbial community composition has been shown to vary across continents and in traditional versus Westernized societies, few studies have examined urban-rural differences in neighboring communities within a single country undergoing rapid urbanization. In this study, we compared the gut microbiome, plasma metabolome, dietary habits, and health biomarkers of rural and urban people from a single Chinese province.

**Results:**

We identified significant differences in the microbiota and microbiota-related plasma metabolites in rural versus recently urban subjects from the Hunan province of China. Microbes with higher relative abundance in Chinese urban samples have been associated with disease in other studies and were substantially more prevalent in the Human Microbiome Project cohort of American subjects. Furthermore, using whole metagenome sequencing, we found that urbanization was associated with a loss of microbial diversity and changes in the relative abundances of Viruses, Archaea, and Bacteria. Gene diversity, however, increased with urbanization, along with the proportion of reads associated with antibiotic resistance and virulence, which were strongly correlated with the presence of *Escherichia* and *Shigella.*

**Conclusions:**

Our data suggest that urbanization has produced convergent evolution of the gut microbial composition in American and urban Chinese populations, resulting in similar compositional patterns of abundant microbes through similar lifestyles on different continents, including a loss of potentially beneficial bacteria and an increase in potentially harmful genes via increased relative abundance of *Escherichia* and *Shigella*.

**Electronic supplementary material:**

The online version of this article (10.1186/s40168-017-0338-7) contains supplementary material, which is available to authorized users.

## Background

As the global population expands and develops, increasing numbers of individuals are transitioning from traditional rural to Westernized urban lifestyles. These changes are associated with increased crowding, altered diet, declines in physical activity, and increased risk of diseases such as obesity, diabetes, cardiovascular disease, and cancer [1–3]. However, the effect of urbanization on the microbiome, the set of microorganisms that lives on or in a person, and how this affects disease risk, has not been explored in detail.

The microbiome plays a crucial role in host function. Dysbiosis of the microbiota has been associated with a number of diseases, including inflammatory bowel disease, diabetes, obesity, and cancer [4–7], which are also associated with a Western lifestyle. Previous studies have shown some modest associations between the microbiome and diet [8–11]. However, in all of these studies, which focused on subjects living similar lifestyles in the same geographical regions, the changes in microbial composition did not overcome inter-individual variation. In contrast, differences in microbial composition become much more pronounced when comparing geographically and culturally distant populations [12–15]. Few studies have addressed urban-rural differences in the microbial and metabolic composition of individuals living within a similar geographic area who share similar ethnicity and diet. This is especially true of recently urbanizing environments. Here, we look at the early stages of how the microbial community evolves in response to recent urbanization.

China is an ideal place to analyze the complex interaction of microbiome, disease, and urbanization, as many areas within this country have only recently (in the past 20 years) started to undergo massive urbanization, and within a single region, one can still find a mix of traditional rural and more Westernized urban lifestyles [16]. This is particularly true in the predominantly Han region of Southern China, which has large variation in timing of major urbanization-related changes. In this study, we analyzed the microbiome and metabolome of 40 subjects from Hunan province, 20 recently urbanized subjects, and 20 rural subjects. By focusing on urban-rural differences in the microbiota and metabolites of a single province, we were able to capture early alterations in response to urbanization, as well as eliminate some of the confounding factors in previous studies, such as ethnicity, geography, and regional dietary patterns. Our results suggest the possibility of a loss of beneficial microbes associated with urbanization, with a concomitant increase in potentially dangerous genes, leading to a more Westernized microbiome.

## Results

### Urbanization is associated with a shift in microbial community composition towards commonly observed Western gut microbes

We compared 20 urban subjects from a city of 7 million to 20 rural subjects living in a small village in Hunan province. The urban sample had a higher disposable income and population but lower birth and population growth rate (Table 1). Furthermore, the urban subjects had higher body mass index (BMI), waist circumference, and insulin levels, consistent with studies showing an association between urbanization, obesity, and diabetes [17].

**Table 1:** Characteristics of the populations studied

	Rural	Urban	Adjusted *P* value
Community characteristics			
Population	26,563	7,000,000	
Persons per square kilometer	668	13,150	
Average disposable income	$800	$5600	
Birth rate per 1000	11.7	8.7	
Population growth rate per 1000	5.8	4.5	
Subject characteristics			
Total number subjects	20	20	
Number (%) female	5 (25%)	13 (65%)	
Mean (std) age range	50.6 (11.7)	58.9 (10.99)	
Significant differences in metadata			
Insulin (μU/mL)	4.31	9.84	0.0003
Waist circumference (cm)	73.78	85.03	0.0104
BMI (kg/m2)	21.09	24.45	0.0104
HbA1c (%)	5.65	6.63	0.0147

The mean of the community characteristics and metadata is shown. Only metadata variables significant after correction for multiple hypothesis testing are shown. See Additional file 6: Table S2H for all metadata variables tested

**Fig. 1:**
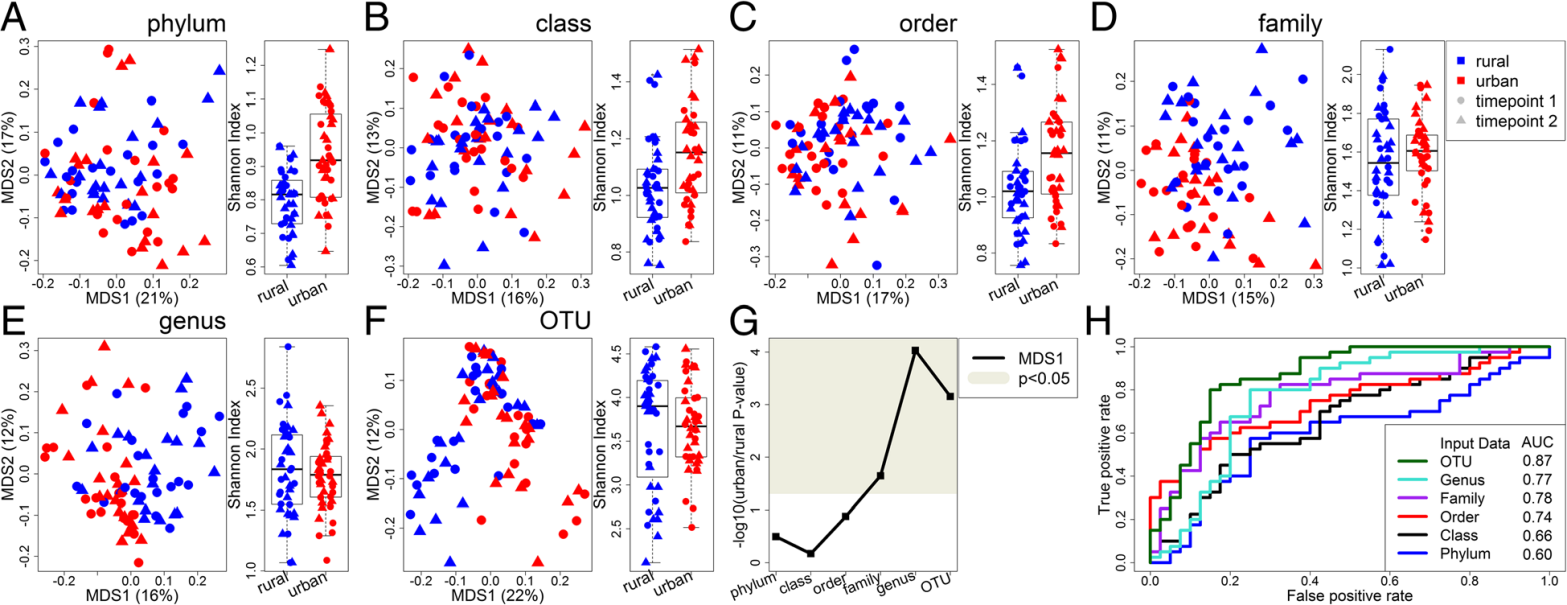
Differences in microbial composition between urban and rural Chinese subjects. **a-f** PCoA plot and comparison of Shannon diversity index for each taxonomic level, based on sequencing the 16S rRNA gene. Points are colored by urban/rural status and shaped by timepoint (each subject gave two samples, separated by 2 weeks). Key is in the top right. Numbers in parentheses on the PCoA axes indicate the percent variation explained by that axis. **g** *P* values comparing the position on the first PCoA axis of urban and rural samples. The gray region indicates significant *P* values (*p* < 0.05). **h** ROC curve using leave-one-out predictions of urban or rural status using a random forest classifier. The area under the curve (AUC) is indicated in the legend

We used 16S ribosomal RNA (rRNA) sequencing of fecal samples to assess the gut microbial community of our subjects at two timepoints, separated by 2 weeks (Fig. 1, Additional file 1: Table S1). Similar to others [18], we found strong correlations between the samples taken at the two timepoints (Additional file 2: Figure S1). Using mixed linear models with urban/rural and time as fixed effects and subject id as a random effect, we found significant differences associated with urban vs. rural status in the first multidimensional scaling (MDS) axis at the operational taxonomic unit (OTU), genus, and family level (Fig. 1g). These differences became much less pronounced at the higher phylogenetic levels of phylum, class, and order. Our observation of increased separation at lower phylogenetic levels is supported by using a random forest classifier (Fig. 1h), which showed that when predicting urban vs. rural status, the area under the curve (AUC) increased with more refined taxonomic levels, with an AUC of over 70%, starting with the family level, suggesting that microbiome composition as measured by 16S rRNA sequencing can be used to differentiate urban and rural subjects at the family level or lower taxonomic levels. In addition, these findings are independent of method and could be reproduced using alternative tools for taxonomic assignment (Additional file 3: Figure S2) and classification (Additional file 4: Figure S3). Intriguingly, while the urban samples had more diversity at the higher taxonomic levels (Fig. 1 and Additional file 5: Figure S4), richness trended opposite to the diversity measures (Additional file 5: Figure S4B), suggesting that this difference is driven by differences in evenness. Taken together, our results suggest that there are significant differences between rural and urban samples that overcame inter-individual variation, but become much less pronounced at higher taxonomic levels.

**Fig. 2:**
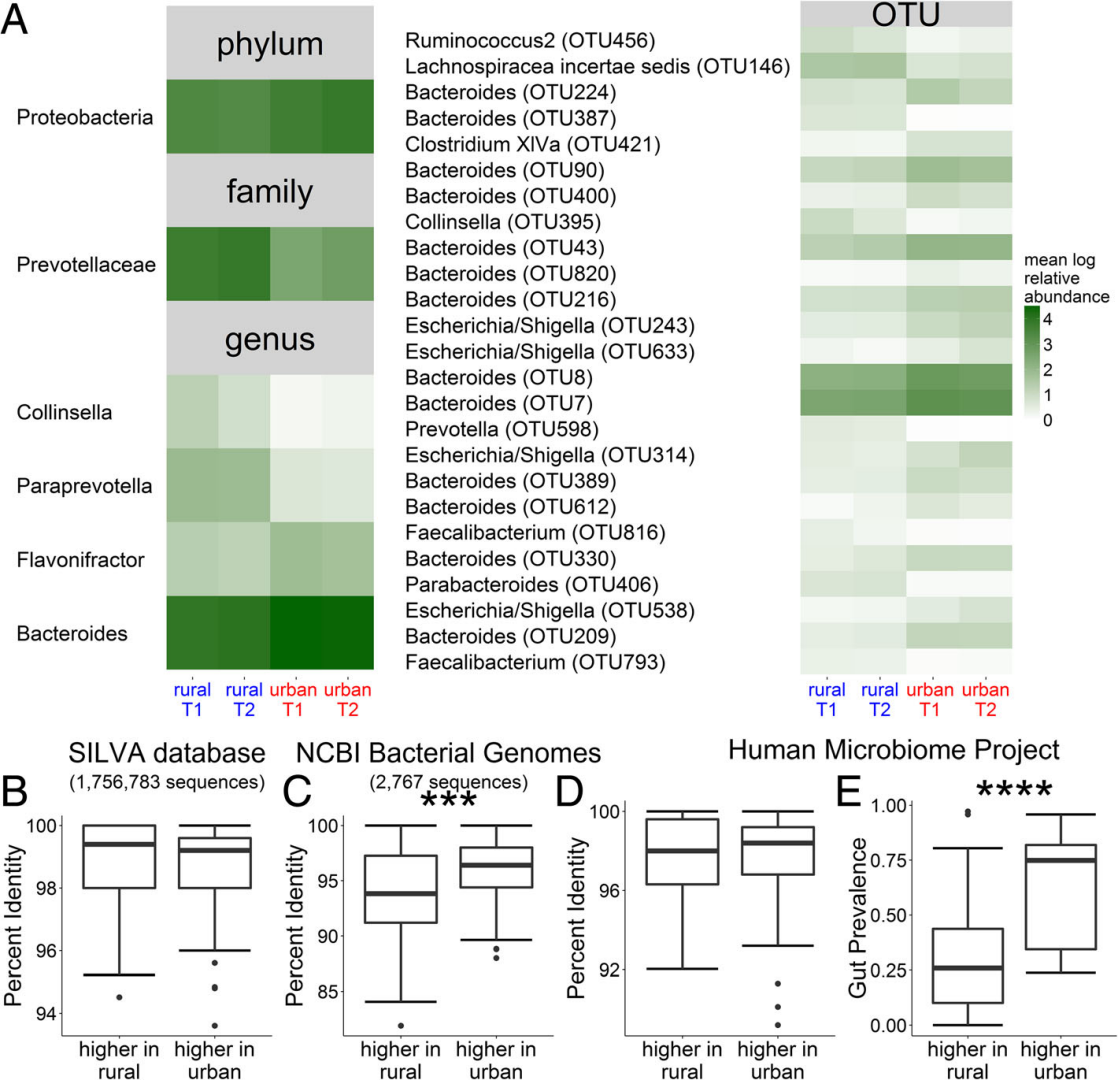
The urban Chinese microbiome is significantly more prevalent in an American cohort than the rural Chinese microbiome. **a** Heatmap showing the mean log normalized relative abundance from sequencing the 16S rRNA gene at timepoints 1 (T1) and 2 (T2) of taxa significantly different (adjusted *p* < 0.05) between urban and rural samples after correction for multiple hypothesis testing, based on our mixed linear models with urban/rural and time as fixed effects and subject id as a random effect. Within each taxonomic level, taxa are given from most to least significant between urban and rural samples. No taxa were significant at the class or order level. At the OTU level, the genus and consensus number is given. At all other levels, the name of the taxon is given. 8 taxa were tested at the phylum level, 16 at the class level, 22 at the order level, 40 at the family level, 86 at the genus level, and 703 at the OTU level. Full details for each taxon, including mean and standard deviation, *P* values and model *R* ^2^ are given in Additional file 6: Table S2A–F. **b-e** We used the consensus sequences from the 80 OTUs higher in rural samples and 91 OTUs higher in urban samples identified from our mixed linear models (unadjusted *p* < 0.05) as query sequences against different 16S rRNA databases. **b** OTUs higher in rural and urban communities are present in the SILVA database of full-length 16S rRNA sequences at similar levels of identity (*p* = 0.08 by unpaired Wilcoxon test). **c** OTUs higher in urban subjects are better represented in the NCBI collection of finished bacterial genomes than OTUs higher in rural subjects (*p* = 0.0008). **d** OTUs higher in rural and urban communities have previously been found in the Human Microbiome Project (HMP), a study of healthy American subjects, at similar levels of identity (*p* = 0.88), but (**e**) OTUs higher in urban subjects are seen in a greater proportion of HMP subjects compared to OTUs higher in rural subjects (*p* = 7.9 × 10^−14^). ***p < 0.001, ****p < 10^−13^

Using our models, there was one phylum, one family, four genera, and 25 OTUs significantly different between rural and urban subjects after correcting for multiple hypothesis testing (Fig. 2a; Additional file 6: Table S2). To further characterize differences between the rural and urban samples, we used the consensus sequences generated by our de novo OTU pipeline as query sequences to search databases of 16S rRNA sequences. Using an unadjusted (uncorrected for false discovery rate) threshold of *p* < 0.05, we mapped the 80 consensus sequences representing OTUs with higher relative abundance in rural samples and the 91 OTUs with higher relative abundance in urban samples to the SILVA database, which contains ~ 1.7 million known 16S rRNA sequences [19] (Fig. 2b). For both rural and urban OTUs, the percent identity to a previously identified sequence was > 99% and there was no significant difference between the percent identities in the taxa higher in rural or urban samples (*p* > 0.05 by the unpaired Wilcoxon test). In contrast, taxa higher in urban samples were significantly (*p* < 0.001) better represented among the 2767 finished bacterial genomes that have been deposited at NCBI (National Center for Biotechnology Information) Genome (Fig. 2c). Thus, although we found little evidence of novel taxa, the genomes of taxa higher in urban subjects have been better characterized by whole genome sequencing projects.

In order to compare our Chinese cohort to a Western cohort, we mapped taxa higher in rural or urban samples to their nearest taxa as represented by consensus sequences from the Human Microbiome Project (HMP), which analyzed the microbiomes of healthy subjects living in the United States [20]. Similar to our findings with the SILVA database, we found no significant difference between how well taxa higher in rural or urban samples could be mapped to the HMP taxa (Fig. 2d). However, there was a substantial difference (*p* < 10^−13^) between how prevalent the closest matching taxa within the HMP were (Fig. 2e). The nearest matching HMP taxa was present in an average of 30.1% of HMP subjects for taxa higher in rural samples, compared to 63.7% for taxa higher in urban samples. This suggests that urbanization in China is associated with an increase in microbes that are present in higher numbers of people in Western (urbanized) America. Furthermore, we found differences in prevalence at *P* values above our 0.05 threshold, suggesting that a larger study would have resulted in more OTUs significantly different between rural and urban than the 25 OTUs we reported (Additional file 7: Figure S5).

### The metabolome is strongly correlated with urbanization

**Fig. 3:**
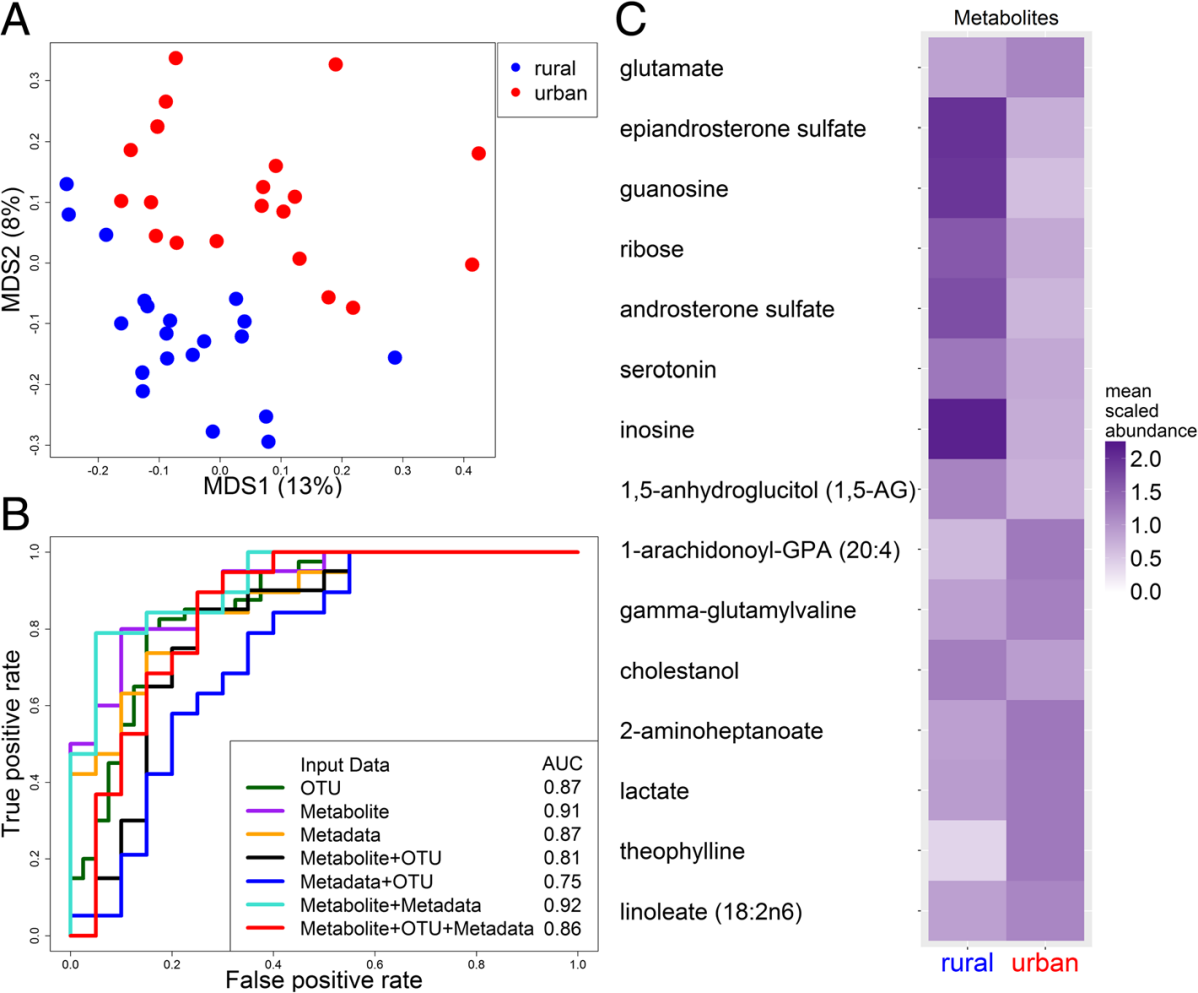
Differences in metabolites between urban and rural Chinese subjects. **a** PCoA plot comparing metabolites between urban (red) and rural (blue) samples. Numbers in parentheses indicate the percent variation explained by that axis. The urban/rural *P* value was 0.02 for MDS1 and 1.5 × 10^−7^ for MDS2. **b** ROC curve using leave-one-out predictions of urban or rural status using a random forest classifier. The area under the curve (AUC) is indicated in the legend. **c** Heatmap showing the mean scaled abundance of metabolites statistically significantly different between urban and rural samples after correction for multiple hypothesis testing (adjusted *p* < 0.05). Metabolites are given from most to least significant. 334 different biochemicals were tested (see Additional file 6: Table S2G for more details, including mean and standard deviation, *P* values and model *R* ^2^)

Metabolites are another sensitive marker of the host’s state [21]. In fact, previous studies have found that the metabolome changes more rapidly than the microbiome in response to host perturbations, such as diet change, suggesting that early response to host changes is transmitted through changes in microbial gene expression more than microbial composition [10, 22]. Due to our finding that the microbiome was stable across timepoints, we generated metabolite data corresponding to the first timepoint from our analysis of the microbiome. We found significant differences between urban and rural metabolite composition (Fig. 3a) that were independent of analysis method (Additional file 8: Figure S6), although there was no difference in the diversity of the metabolome. Furthermore, our machine learning classifier was even better at predicting urban/rural status from the metabolome than the microbiome (Fig. 3b). At an FDR-adjusted *P* value of *p* < 0.05, there were 16 metabolites significantly associated with urban vs. rural status (Fig. 3c; Additional file 6: Table S2G). Ten of the 16 significantly different metabolites were involved in lipid, carbohydrate, and energy metabolism—all key differences in Western (urban) versus traditional Chinese (rural) diets—and a smaller proportion related to peptides, nucleotides, and amino acids, with one xenobiotic (theophylline). Thus, the metabolome appears to respond to urbanization and Western diet even more robustly than the microbiome.

In addition, we collected metadata on diet and other variables, such as anti- or probiotic use, anthropometric measures, arsenic exposure, and several biomarkers for cardiometabolic disease (Additional file 6: Table S2H). These data were also able to relatively accurately predict urban/rural status (Fig. 3b, Table 1), even though significant (adjusted *P* values <0.05) urban/rural differences were only seen in the diabetes and obesity measures. These measures were all higher in urban subjects, suggesting that the risk of diabetes and obesity has increased in our urban population. Of note, none of the diet variables (taken as an average over 3 days) was significantly different between the two groups.

Intriguingly, combining the microbiome data with the metadata or metabolite data decreased the ability of the classifier to distinguish between urban and rural populations, while combining the metadata and metabolite data gave an AUC of 92% (Fig. 3b), the highest out of all other combinations of the microbiome, metabolome, and metadata. Likewise, there were few associations between the microbiome and metadata or metabolome. For example, only five metadata variables were associated with any taxa: antibiotic use in the last 6 months (associated with 18 taxa), use of probiotic supplements (associated with three OTUs), insulin levels (associated with two OTUs), and gender and fat intake (each associated with one OTU; Additional file 9: Table S3). In contrast, there were 133 associations between the metadata and metabolome (Additional file 9: Table S3C). A few of these correlated variables measured the same value in different ways, such as glucose, which was measured both as an independent blood test in the metadata and as a metabolite in our metabolomics data (Additional file 10: Figure S7). This strong correlation in the variables measured in two independent ways validates our metabolomic methods. The majority of the statistically significant associations between the metabolites and the metadata, however, revealed intriguing novel associations between the metabolome and health. For example, among our 133 statistically significant associations, 106 (80%) fell into the amino acid, carbohydrate, lipid, and peptide superpathways. The eight metabolites associated with BMI, waist or weight were all in the peptide, amino acid, or carbohydrate superpathways. The 23 metabolites associated with glucose, HbA1c, and insulin largely fell in glucose metabolism pathways, while the 17 metabolites associated with cholesterol, triglycerides, and LDL largely fell in lipid metabolism pathways.

### The relative abundance of Viruses and Archaea is decreased in urban subjects

**Fig. 4:**
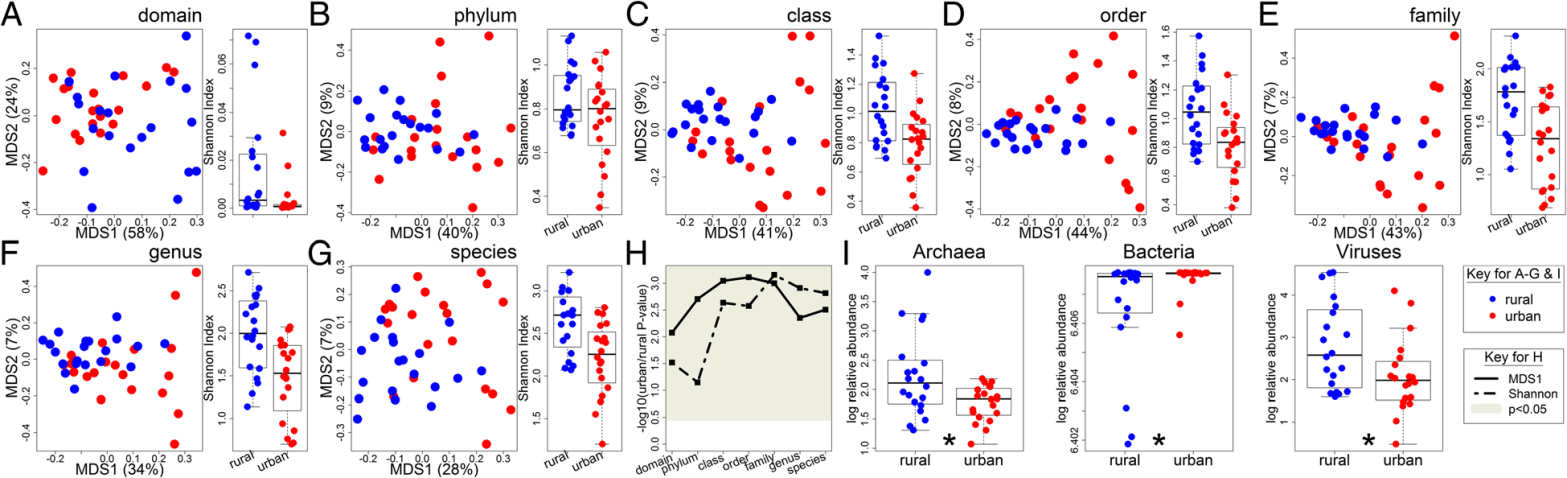
Viruses and Archaea are less abundant in the urban Chinese microbiome while Bacteria are more abundant. **a–g** PCoA plot and comparison of Shannon diversity index for each taxonomic level, based on whole genome sequencing. Blue points indicate rural subjects while red points indicate urban subjects. Numbers in parentheses on the PCoA axes indicate the percent variation explained by that axis. **h** *P* values comparing the position on the first PCoA axis (solid line) or Shannon diversity (dashed line) of urban and rural subjects. The gray region indicates significant *P* values (*p* < 0.05). **i** Comparison of the logged relative abundance of each domain detected using whole genome sequencing. An asterisk (*) in **i** indicates a Benjamini-Hochberg-adjusted *P* value of less than 0.05

While 16S rRNA sequencing can provide valuable insights into which bacteria are present in the microbiome, whole genome sequencing can provide a more complete taxonomic and functional representation of the microbial community. We therefore also performed whole metagenome sequencing of the samples from the first timepoint, generating 10 million reads per sample (Additional file 1: Table S1). These data confirmed the significant difference in microbial composition seen in the lower taxonomic levels by 16S rRNA sequencing (Fig. 4). However, with whole genome sequencing, we saw separation between urban and rural samples at all taxonomic levels, including domain. This increased separation at the higher taxonomic levels may be due to the additional taxa detected at each level with the more sensitive whole genome sequencing (Additional file 11: Table S4).

In addition, we found that at every taxonomic level urban subjects had decreased diversity, consistent with other studies showing loss of diversity with Westernization [12, 13]. This was also true for other diversity measures (Additional file 12: Figure S8), but is opposite what was seen for our 16S rRNA Shannon diversity measures, where urban subjects had higher diversity at the upper taxonomic levels, with no significant difference or lower diversity at the lower taxonomic levels (Fig. 1 and Additional file 5: Figure S4). However, richness from the 16S rRNA analysis was decreased in urban samples (Additional file 5: Figure S4B), similar to the whole genome sequencing results. Our data are consistent with the hypothesis that at higher phylogenetic levels changes in evenness in a few dominant taxa drive our observed differences in 16S diversity. These dominant taxa may be a result of 16S rRNA sequencing bias. In contrast, there appear to be fewer dominant taxa in the whole genome sequencing data, which detected many more taxa at each phylogenetic level compared to 16S rRNA sequencing (Additional file 11: Table S4), and provides a more consistent view of decreased diversity with urbanization across all phylogenetic levels, no matter which diversity measurement is used.

Using our linear models on the taxonomic data generated from our whole metagenome sequencing, we detected 60 species, 44 genera, 71 families, 35 orders, 21 classes, 9 phyla, and 3 domains significantly different between urban and rural subjects after correcting for multiple hypothesis testing (Additional file 13: Table S5A-G). Most of these taxa were more abundant in rural subjects, further supporting the hypothesis that urbanization is associated with a loss of potentially beneficial bacteria (Additional file 14: Figure S9). Interestingly, all three domains detected (Bacteria, Archaea, and Virus) were significantly different between urban and rural subjects (Fig. 4i), suggesting that urbanization results in an overall loss of Archaea and Viruses and a very small but significant increase in the relative abundance of Bacteria. Archaea are an understudied part of the microbiome, but are generally methanogens. They have been associated with leanness and archaebiotics have been proposed to modulate the risk of atherosclerosis [23, 24]. Likewise, the virome has not been as well characterized, and although many well-known viruses cause disease, some viruses can replace the beneficial functions of commensal bacteria [25, 26].

As with the 16S rRNA sequencing results, there were a number of intriguing associations between the relative abundance of taxa and metabolites (Additional file 15: Table S6A). For example, 10-undecenoate (11:1n1) was positively associated with the family Leuconostocaceae or other taxa belonging to this family in both 16S rRNA and whole genome sequencing. Leuconostocaceae are lactic acid bacteria previously shown to undergo niche-specific evolution [27] and are associated with ulcerative colitis [28], while 10-undecennoate (11:1n1) has been associated with colorectal cancer [29]. In contrast, there were only 16 associations between taxa and the metadata: most were associations with antibiotic use in the past 6 months; although the Archaea domain was associated with sodium intake, the *Melisococcus* genus and one of its species was associated with probiotic supplements, and one phage was associated with HDL (high density lipoprotein cholesterol; Additional file 15: Table S6B). Thus, urban/rural status is strongly associated with microbial composition as measured by whole-genome shotgun sequencing, confirming our observations from sequencing the 16S rRNA gene, including dysbiosis in the relative abundance of entire taxonomic domains.

### Urban subjects have increased gene diversity, including increased antibiotic resistance

**Fig. 5:**
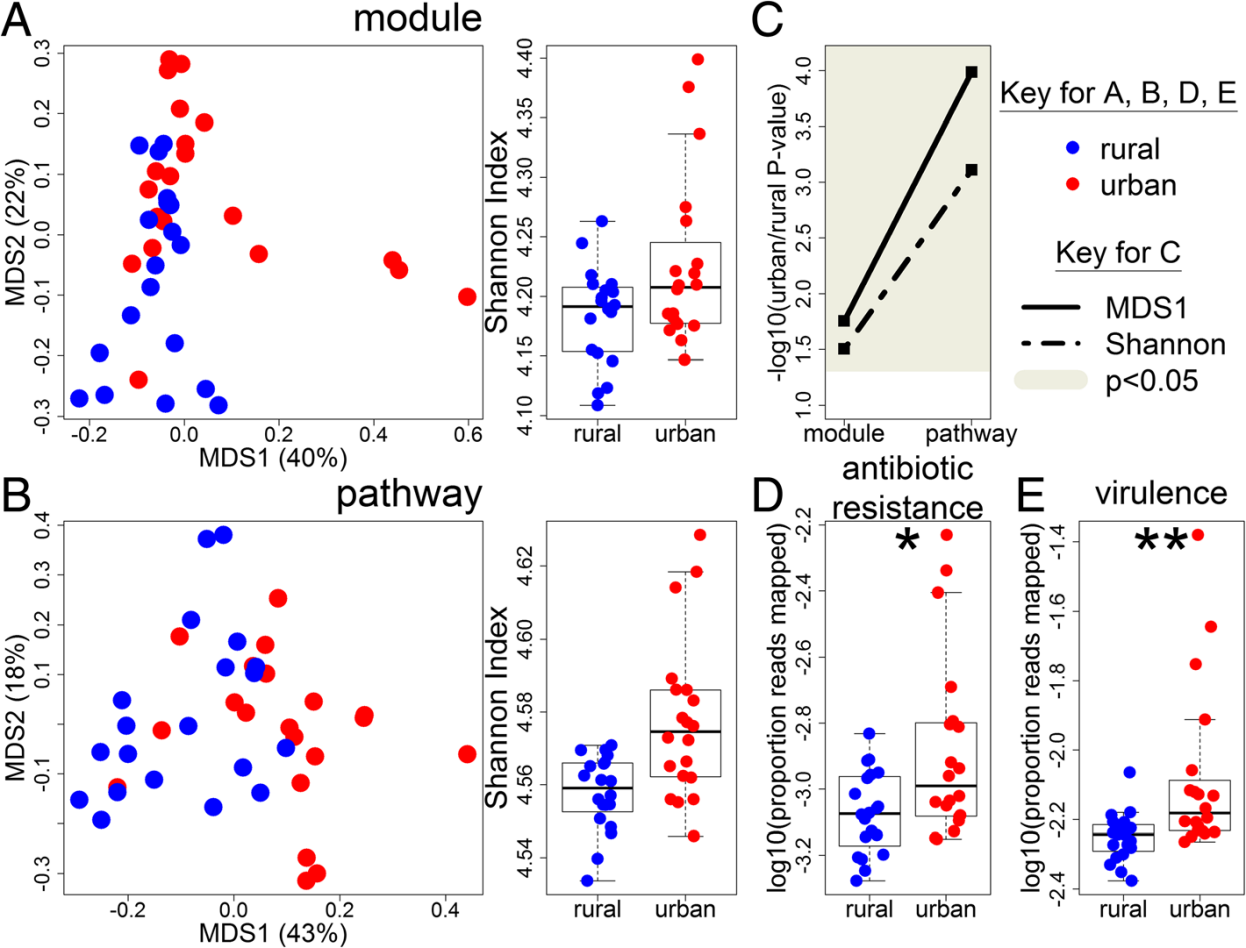
The microbiome of urban Chinese subjects encodes more genetic diversity, including more genes conferring antibiotic resistance, than rural Chinese subjects. **a** PCoA plot and comparison of Shannon diversity index for KEGG modules and **b** KEGG pathways. Blue points indicate rural subjects while red points indicate urban subjects. Numbers in parentheses on the PCoA axes indicate the percent variation explained by that axis. **c** *P* values comparing the position on the first PCoA axis (solid line) or Shannon diversity (dashed line) of urban and rural subjects. The gray region indicates significant *P* values (*p* < 0.05). **d–e** Comparison of the proportion of reads from whole genome sequencing that aligned to (**d**) the CARD protein homolog database [31] or **e** MvirDB [32]. The asterisk (*) in **d** indicates a *P* value of less than 0.05 comparing urban and rural subjects (*p* = 0.01), while two asterisks (**) in **e** indicates a *P* value of less than 0.01 (*p* = 0.006)

We also used our whole genome sequencing data to analyze the relative abundance of KEGG modules and pathways (Fig. 5). Again, we saw a significant separation between urban and rural samples, indicating that the gene content is changing with the altered microbial composition. Intriguingly, the urban samples had increased diversity compared to rural samples, opposite the results seen from the whole genome sequencing taxonomic results, which were generated from the same reads (Figs. 4 and 5, Additional file 12: Figure S8 and Additional file 16: Figure S10). Given this finding, for each OTU, we looked at the number of annotated genes contained in the closest finished NCBI bacterial genome (Fig. 2c). We found that the taxa higher in urban samples also contained more annotated genes than the taxa higher in rural samples (Additional file 17: Figure S11). Taken together, this suggests that urban subjects have lost diversity in terms of microbial composition, but the taxa that are present encode more KEGG functions.

There were 10 modules and 51 pathways significantly different between urban and rural subjects, many of which were involved with metabolism, helping to confirm our metabolite findings (Additional file 13: Table S5H-I). For example, M00048 (inosine monophosphate biosynthesis) was one of the modules significantly more abundant in rural samples while the metabolite inosine was also significantly higher in rural samples. Likewise, ko00280 (valine, leucine, and isoleucine degradation) was significantly lower in rural samples, while the metabolite gamma-glutamylvaline, which is likely a short-lived intermediate from protein degradation on the way to specific amino acid degradation, was also significantly lower in rural subjects, suggesting a decrease in valine degradation [30]. While the majority of these KEGG functions were more abundant in rural samples, the proportion of significant functions higher in rural was not as pronounced as in the taxonomic results (Additional file 18: Figure S12). Twenty-three modules and 4 pathways were associated with our metadata, including many of the three-day dietary measures, while 139 modules and 9 pathways were associated with metabolite data, suggesting a stronger interaction between gene content, diet, and metabolites than what was seen with the taxonomic data (Additional file 15: Table S6C-D).

One set of genes of particular interest when comparing urban versus rural participants is the genes encoding antibiotic resistance. To analyze the presence of antibiotic resistance in the microbiome, we mapped all reads from our whole genome sequencing analysis to the comprehensive antibiotic resistance protein homolog database (CARD) [31]. Genes in this database are genes whose presence confers antibiotic resistance, such as β-lactamases. We found that significantly more reads from urban subjects mapped to the database than rural subjects (Fig. 5d), suggesting that urbanization is associated with increased antibiotic resistance, even though antibiotic use was not different between the two groups (no subject had taken antibiotics in the past 3 months and only three had taken antibiotics in the past 6 months, two of which were rural and the other urban). Of the three urban samples with the highest rates of antibiotic resistance genes, only one had taken antibiotics in the past 6 months. Furthermore, if we remove the three urban samples with the highest proportion of reads mapped to CARD, we still get a *P* value of 0.05, suggesting these results are not driven entirely by these three points. Taken together, our results may have important implications for the efficacy of antibiotics in treating infections in the urban population, as many antibiotic resistance genes are encoded on plasmids and easily move between bacterial strains. Thus, some of the increased gene content in urban subjects encodes genes that could potentially harm the host.

When we looked for associations between the proportion of reads that mapped to antibiotic resistance genes and our microbiome, metadata, and metabolite data, we found no significant associations between the metabolite data or metadata. In contrast, 80 KEGG modules and 135 KEGG pathways were significantly associated with the proportion of reads that mapped to antibiotic resistance genes (Additional file 19: Table S7). In considering the associations between the proportion of antibiotic resistant reads and taxonomy, we found that most of the significant associations with the proportion of reads that mapped to antibiotic resistance genes were with *Escherichia* and *Shigella* in both our 16S rRNA and whole genome sequencing data (Additional file 20: Figure S13; Additional file 19: Table S7A–B). Intriguingly, a significant association appeared in all OTUs or species classified as belonging to these genera, and in all higher taxonomic levels that these genera belong to. Many of these taxa were significantly higher in urban subjects in the 16S rRNA analysis and trended towards higher relative abundance in the whole genome sequencing data. Taken together, these data demonstrate associations between urbanization, an increased relative abundance of *Escherichia* and *Shigella* and an increased proportion of reads mapping to antibiotic resistance genes*.* Our data are therefore consistent with a hypothesis that during urbanization, these genera become more abundant, bringing with them genes that result in increased antibiotic resistance.

In order to further assess the presence of genes associated with virulence and pathogenicity, the whole genome sequencing reads were also aligned to MvirDB [32], a database that combines genes associated with virulence factors, toxins, and pathogen-host interactions. As with the CARD database, we found that a significantly higher proportion of reads aligned to MvirDB in urban subjects than rural subjects (Fig. 5e). This was true even after removing the three urban samples with the highest proportion of mapped reads. In addition, similar to the CARD findings, there was a strong association between the proportion of reads mapped to MvirDB and *Escherichia* and *Shigella* (Additional file 21: Table S8). Taken together, these data suggest a model in which urbanization results in a Westernization of the gut microbiome, resulting in loss of taxonomic diversity but increase in gene diversity, including loss of Viruses and Archaea but increased antibiotic resistance and virulence, suggesting a loss of microbes beneficial to the host with a gain in potentially deleterious genes, which may be caused by an increase in *Escherichia* and *Shigella.*

## Discussion

We have found significant differences between rural and urban subjects in both microbiome community composition, as measured by both 16S rRNA sequencing and whole genome sequencing, and microbiome gene content. In particular, urbanization was associated with decreased microbial diversity, including a loss of Viruses and Archaea. This dysbiosis in non-bacterial domains is highlighted by the fact that differences between urban and rural subjects as measured by 16S rRNA sequencing, which can only capture Bacteria, did not propagate above the family level. This contrasts previous studies that found pronounced microbiome differences at all taxonomic levels, even using 16S rRNA sequencing. However, those studies compared samples from different continents, genetic backgrounds, and social practices [12–15]. In fact, studies focusing on urban and rural differences within Asia found that the microbiome clusters strongly by ethnicity and geography and not by social and dietary lifestyles [33, 34]. Our focus on two neighboring Han communities enabled us to control for differences in ethnicity, geography, and regional dietary patterns and enabled us to find differences even in our recently urbanized populations.

We have done our best to control for diet, antibiotic use, and other potentially confounding factors by showing that our metadata is not significantly different between the two populations, but there always remains the chance that there is some additional variable that can contribute to urban-rural differences that a larger study will be able to identify. Our study made extensive utilization of linear models; however, our linear model *P* values are highly correlated with our nonparametric tests (Additional file 22: Supplemental Text; Additional file 23: Table S9), indicating that lack of normality is not what is driving our results. Given our small sample size of 20 subjects per group, we utilized FDR correction separately for each taxonomic level and did not pool hypotheses across our metabolite, 16S and whole-genome sequencing results for correction. This strategy is anticonservative and increases the chance that some of the associations we report with individual taxa, genes, and metabolites may be the results of overfitting. However, we also identified strong differences between urban and rural community compositions that do not require extensive correction for multiple hypotheses, such as analysis of PCoA axes and the results of machine learning algorithms. So while the results we report for individual taxa, metabolites, and genes will ultimately require validation in a larger cohort, we are confident that the overall differences we report between rural and urban populations are not simply a result of our multiple hypothesis correction strategy. Furthermore, a recent observation in mice found that changes in microbial composition in response to altered diet increase with each generation [35], so we predict that as we continue to sample these urbanizing populations over time, the microbial differences will become more pronounced. Thus, our study may represent an observation of the early steps towards a fully Westernized gut microbial composition in China.

Of particular interest is our finding of increased relative abundance of genes that confer antibiotic resistance in urban samples, despite the fact that in our study antibiotic use in the past 6 months was not significantly different between urban and rural subjects, and no study participant had used antibiotics in the last 3 months. Our results suggest that changes associated with urbanization may indirectly increase the reservoir of resistant genes and virulence factors via increased colonization by pathogenic *Escherichia* and *Shigella*. Virulence factors encoded by *Escherichia* have previously been associated with diseases such as colorectal cancer [36] and Crohn’s disease [28]. Thus, the complex interplay between disease, urbanization, and the microbiome is an important topic for future studies.

In terms of composition, taxa that were more abundant in rural samples were also much less prevalent in the American cohort studied in the HMP. A natural hypothesis is that these taxa—higher in rural China but of lower prevalence in America and poorly represented in finished genome sequencing projects—represent a beneficial set of microbes that are suppressed but not eliminated by urbanization. Our study therefore suggests the possibility that Westernization of the microbiome drives disease by increasing the prevalence of pathogens, such as *Escherichia/Shigella*, that harbor harmful characteristics, such as antibiotic resistance genes and virulence factors, and by reducing the relative abundance of beneficial taxa. Our compositional findings suggest that since these taxa are higher in our rural cohort and are not entirely absent in Western populations, changes in lifestyle could presumably allow them to again become more prevalent, potentially increasing host health. Likewise, our study suggests these taxa as potential targets of probiotics. For example, several *Ruminococcus* species were significantly more abundant in rural samples, and the most significant OTU from 16S rRNA sequencing also belonged to this genus. This genus has been associated with diet and the breakdown of resistant starch and also produces butyrate, which has previously been shown to play an important role in immune function [37–39]. Establishing the validity of the hypothesis that the partial Westernization of the microbiome helps to drive disease will ultimately require long-term longitudinal surveys. Executing these studies is particularly vital as these rural populations begin to urbanize or migrate and disappear.

## Conclusions

We compared the gut microbiome and plasma metabolome of rural and urban people from a single Chinese province and found significant differences in the composition of recently urbanized and rural subjects. These changes include an alteration in the relative abundances of entire domains, particularly loss of Archaea and Viruses with urbanization. Furthermore, our analysis of the composition of the microbiome suggests that the microbes that had a higher relative abundance in the urban Chinese samples also had a higher relative abundance in an American cohort. In addition, the genes encoded by the microbiome and the metabolites generated have significantly altered with urbanization, with an increase in overall genetic diversity as well as in genes associated with resistance and virulence. Our results suggest that urbanization is rapidly altering the gut microbial community, making it more similar to a Western composition, resulting in higher levels of antibiotic resistance and virulence genes. These changes are accompanied by an increase in biomarkers of diseases traditionally associated with Westernization, such as diabetes, obesity, and cancer. Taken together, our study suggests a hypothesis that urbanization/Westernization results in rapid convergent evolution across the globe of the microbial community to a dysbiotic state, with an increase in the prevalence of pathogens that harbor harmful genes and a concomitant reduction in the relative abundance of beneficial taxa.

## Methods

A random sample of individuals from Hunan Province (in Southern China) aged 18–65 were selected for the study, with exclusion of individuals who lived in these communities for less than 2 years, were pregnant or lactating, had known cancer, worked in an occupation with potential exposure to arsenic, or had antibiotic use within the past 3 months. Metadata, fasting blood, spot urine, and fecal samples were collected by China CDC data collectors during a three-day house visit, with a repeat fecal collection 2 weeks later. Metabolites were assayed using Chromatography/Mass Spectrometry (Metabolon, Inc.). Sequencing was performed by the Beijing Genomics Institute-Shenzhen, using an Illumina MiSeq PE250 for 16S sequencing targeting the V4 hypervariable region (16S rRNA sequencing) or an Illumina HiSeq 4000 (whole metagenome sequencing). 16S rRNA sequencing reads were classified using the RDP (Ribosomal Database Project) classifier [40]. Kraken [41] was used to assign taxonomy to the whole genome sequencing reads while HUMAnN [42] was used to assign KEGG functions. In addition, whole genome sequencing reads were aligned to the Comprehensive Antibiotic Resistance Database (CARD) protein homolog database, version 1.0.4 [31] or to MvirDB [32] to assess the presence of antibiotic resistance and virulence factors. All sequences have been deposited in NCBI SRA (https://www.ncbi.nlm.nih.gov/sra) under BioProject PRJNA349463. Accession numbers are given in Additional file 1: Table S1. *P* values for urban-rural status were calculated using mixed linear models. Nonparametric models were used to confirm these results (Additional file 23: Table S9). See Additional file 22: Supplemental Text for more details.

## Additional files

Additional file 1: Table S1. Sequencing statistics and accession numbers for each sample. (XLSX 14 kb)

Additional file 2: Figure S1. Correlation between timepoints. Comparison between the first timepoint (*x*-axis) and second timepoint (*y*-axis) for the first (first column) and second (second column) axes of the PCoA plots for the 16S rRNA sequencing data at the genus and OTU taxonomic levels. Black line indicates the linear regression model and the number in the lower right indicates the multiple *R* ^2^ of that model. Urban samples are colored red while rural samples are blue. (TIFF 22968 kb)

Additional file 3: Figure S2. Differences between urban and rural microbial composition is not dependent on bioinformatics pathway. (A–F) PCoA plot for each taxonomic level. Microbial composition was determined using the closed reference function of QIIME (as compared to RDP or Abundant OTU+ in Figs. 1 and 2) on the 16S rRNA sequencing data. Number in parentheses is the percent variation explained by that axis. (TIFF 20508 kb)

Additional file 4: Figure S3. Use of an alternative classifier confirms the ability to predict urban/rural status from the microbiome, the metabolome, or metadata. ROC curve generated from predictions from the rpart *R* function, using leave-one-out predictions of urban or rural status. The area under the curve (AUC) is indicated in the legend. (TIFF 46875 kb)

Additional file 5: Figure S4. Differences in microbial diversity based on 16S rRNA sequencing are driven by differences in evenness and are opposite differences in richness. Comparison of (A) inverse Simpson diversity index, (B) richness, or (C) evenness for each taxonomic level, based on 16S rRNA sequencing. *P* values indicate the significance of the difference between urban and rural subjects. Microbial composition was determined using RDP (phylum-genus) or AbundantOTU+ (OTU). (TIFF 34218 kb)

Additional file 6: Table S2. Model results for all microbial taxa identified through 16S rRNA sequencing, metabolites, and metadata. Each tab corresponds to a different set of analyses: (A) phylum, (B) class, (C), order, (D) family, (E) genus, (F) OTU, (G) metabolome, (H) metadata. For the microbiome (A-F), the columns indicate the taxa; full taxonomy (p for phylum, c for class, o for order, f for family, g for genus); mean and standard deviation for rural and urban in timepoint 1, timepoint 2 and both timepoints; linear model *P*-values (unadjusted and Benjamini and Hochberg-adjusted); linear model R^2^; and Wilcoxon rank sum test *P*-values for timepoint 1 and 2 (unadjusted and Benjamini and Hochberg-adjusted). (G-H) The columns indicate the metabolite or metadata name; the super and sub pathway for the metabolite (G only); the mean and standard deviation for rural and urban; the linear model *P*-values (unadjusted and Benjamini and Hochberg-adjusted); and the linear model R^2^. (G) The last two columns are the unadjusted and Benjamini and Hochberg-adjusted *P*-values from a Wilcoxon rank sum test. (H) The last three columns indicate the nonparametric test used (Wilcox: Wilcoxon rank sum test, used for continuous data; Fisher: Fisher’s exact test for categorical data), followed by the unadjusted and Benjamini and Hochberg-adjusted nonparametric test *P*-values for urban vs. rural status. For antibiotic or probiotic use, a 0 indicated no use and a 1 indicated use. For gender, a 1 indicated male and a 2 indicated female. All sections are sorted by *P*-value for urban vs. rural status. (XLSX 392 kb)

Additional file 7: Figure S5. Prevalence across the data set. Urban and rural prevalence vs. unadjusted *P* value for the null hypothesis that the OTU had the same distribution in rural and urban populations. Each point represents the average Human Microbiome Project prevalence of a 25 OTU windows. (TIFF 4218 kb)

Additional file 8: Figure S6. PLS-DA confirms the separation of urban and rural samples using their metabolomic profiles. Score plot for the first two dimensions (*t* [1] and *t* [2]) defined by PLS-DA. Urban (red) and rural (blue) metabolomics profiles were very well separated by PLS-DA with R2Y = 0.963 (which indicates good model fit) and Q2Y = 0.637 (which indicates good predictability). Subject number is labeled on the plot. The separation passed permutation based validation. The predictability of this model was found to be better than any models built using the 999 permutated data sets. (JPEG 885 kb)

Additional file 9: Table S3. Significant associations between microbial taxa identified through 16S rRNA sequencing, metabolites, and metadata. Significant associations (Benjamini and Hochberg-adjusted linear model *P* value < 0.05) between (A) the microbiome as assessed by 16S rRNA sequencing and metabolite data, (B) the microbiome as assessed by 16S rRNA sequencing, and metadata or (C) the metadata and metabolite data. (A–B) p indicates the phylum, c the class, o the order, f the family, and g the genus. Each section is ordered by taxonomic level and then by *P* value. (XLSX 40 kb)

Additional file 10: Figure S7. Variables measured in both the metadata and metabolite analyses are strongly correlated. Correlation between metadata and metabolite results for (A) cholesterol or (B) glucose, which were independently measured in these two analyses. The metadata cholesterol measure includes cholesterol esters while the metabolite analysis does not. The Spearman correlation and the adjusted *P* value were calculated from a linear model of the metabolite vs. metadata values, corrected for multiple hypothesis testing (Additional file 9: Table S3C). The model is indicated with the black line. (TIFF 98437 kb)

Additional file 11: Table S4. Number of taxa and genes identified in analysis. Number of taxa (and genes) identified through 16S rRNA sequencing or whole genome sequencing at each taxonomic level after filtering taxa (or genes) absent from more than one quarter of the samples. (XLSX 10 kb)

Additional file 12: Figure S8. Differences in microbial diversity based on whole genome sequencing is not dependent on diversity index used. Comparison of (A) inverse Simpson diversity index, (B) richness, or (C) evenness for each taxonomic level based on whole genome sequencing. *P* values indicate the significance of the difference between urban and rural subjects. (TIFF 39937 kb)

Additional file 13: Table S5. Model results for whole genome sequencing data. (A–G) All microbial taxa identified through whole genome sequencing: (A) domain, (B) phylum, (C) class, (D), order, (E) family, (F) genus, and (G) species. (H–I) All KEGG functions identified through whole genome sequencing: (H) modules and (I) pathways. Starting with the first column, the columns indicate the taxa name (A–G) or KEGG designation (H–I), the full taxonomy (A–G) or KEGG description (H–I), the mean and standard deviation for all rural samples, the mean and standard deviation for all urban samples, the *P* value from our linear model for urban vs. rural status, the Benjamini and Hochberg-adjusted linear model *P* value, the linear model *R* ^2^, the *P* value from a Wilcoxon rank sum test for urban vs. rural status, and the Benjamini and Hochberg-adjusted Wilcoxon *P* value. All sections are sorted by linear model *P* value for urban vs. rural status. (XLSX 467 kb)

Additional file 14: Figure S9. Taxa significantly different in relative abundance between urban and rural subjects in whole genome sequencing have higher relative abundance in rural subjects. Volcano plots of the adjusted *P* values vs. fold change from whole genome sequencing at each taxonomic level. The *P* values given at the top of each plot were calculated using a chi-squared test. The horizontal dashed gray line indicates an adjusted *P* value of 0.05 while the vertical dashed gray line indicates an urban/rural fold change of 1. See Additional file 13: Table S5A-G for detailed model results for all taxa tested, including mean and standard deviation, *P* values, effect sizes (as measured by model *R* ^2^), and Spearman correlation. (TIFF 21093 kb)

Additional file 15: Table S6. Significant associations between whole genome sequencing results and metabolites or metadata. Significant associations (Benjamini and Hochberg-adjusted linear model *P* value < 0.05) between (A) microbial taxa identified through whole genome sequencing and metabolite data, (B) microbial taxa identified through whole genome sequencing and metadata, (C) KEGG functions and metabolite data, or (D) KEGG functions and metadata. For the microbial taxa (A–B), p indicates the phylum, c the class, o the order, f the family, g the genus, and s the species. Each section is ordered by level and then by *P* value. (XLSX 46 kb)

Additional file 16: Figure S10. Urban subjects have higher gene diversity and evenness but equal richness compared to rural subjects. Comparison of (A) inverse Simpson diversity index, (B) richness, or (C) evenness for KEGG modules (left column) and KEGG pathways (right column), based on whole genome sequencing. *P* values indicate the significance of the difference between urban and rural subjects. (TIFF 11250 kb)

Additional file 17: Figure S11. Taxa more abundant in urban samples have more genes than taxa that are more abundant in rural samples. For each OTU, the number of annotated genes in the closest finished genome in NCBI (Fig. 2c) was counted. The *P* value is from a *t* test comparing the 80 OTU consensus sequences with higher relative abundance in rural samples to the 91 OTUs with higher relative abundance in urban samples. (TIFF 5273 kb)

Additional file 18: Figure S12. KEGG pathways significantly different in relative abundance between urban and rural subjects tend to have a higher relative abundance in rural subjects. Volcano plots of the adjusted *P* values vs. fold change from whole genome sequencing for KEGG modules (left) and KEGG pathways (right). The *P* values given at the top of each plot were calculated using a chi-squared test. The horizontal dashed gray line indicates an adjusted *P* value of 0.05 while the vertical dashed gray line indicates an urban/rural fold change of 1. See Additional file 13: Table S5H-I for all KEGG functions tested and the model results, including mean and standard deviation, *P* values, effect sizes (as measured by model *R* ^2^), and Spearman correlation. (TIFF 9521 kb)

Additional file 19: Table S7. Significant associations with antibiotic resistance. Significant associations (Benjamini and Hochberg-adjusted linear model *P* value < 0.05) between the proportion of reads that mapped to the CARD protein homolog database [31] and (A) microbial taxa identified through 16S rRNA sequencing, (B) microbial taxa identified through whole genome sequencing (WGS), or (C) KEGG modules and pathways. There were no significant associations between the proportion of reads that mapped to the CARD protein homolog database and any metadata or metabolite. Each section is sorted by level and then *P* value. (XLSX 45 kb)

Additional file 20: Figure S13. The proportion of reads that map to genes that confer antibiotic resistance is associated with *Escherichia* and *Shigella*. Correlation between the proportion of whole genome sequencing reads that aligned to the CARD protein homolog database [31] (*y*-axis) to the relative abundance of the indicated taxa in that sample (*x*-axis). (A) The relative abundances were from 16S rRNA sequencing. (B) The relative abundances were from whole genome sequencing. The *Escherichia* and *Shigella* genera are shown, as well as each of the higher taxonomic levels that they belong to. All 14 OTUs classified into these genera were also significant (16S), as well as all six species identified in whole genome sequencing. In the upper left corner of each plot, r is the Pearson correlation coefficient and p is the Benjamini and Hochberg-adjusted *P* value from an ANOVA of the linear model. The model is indicated with the black line. See Additional file 19: Table S7 for further details. Similar patterns were seen with the proportion of whole genome sequencing reads that aligned to MvirDB (Additional file 21: Table S8). (TIFF 35156 kb)

Additional file 21: Table S8. Significant associations with virulence genes. Significant associations (Benjamini and Hochberg-adjusted linear model *P* value < 0.05) between the proportion of reads that mapped to MvirDB [32] and (A) microbial taxa identified through 16S rRNA sequencing, (B) microbial taxa identified through whole genome sequencing (WGS), or (C) KEGG modules and pathways. There were no significant associations between the proportion of reads that mapped to MvirDB and any metadata or metabolite. Each section is sorted by level and then *P* value. (XLSX 45 kb)

Additional file 22: Supplementary Text. Detailed Methods. (DOCX 324 kb)

Additional file 23: Table S9. Data that were significantly different between urban and rural subjects using linear model results were generally also significant when using nonparametric models. The columns indicate the type of data, the phylogenetic, or gene level (or NA if no level is applicable), the number of values significantly different between urban and rural subjects (adjusted *P* value < 0.05) using a linear model, the number significantly different between urban and rural subjects using a nonparametric model, the number of values significant in both models, and then whether MDS1 was significantly different between urban and rural subjects using a linear model, nonparametric model, and in both models. 16S rRNA sequencing data was not included, as we had multiple samples from the same individual at two timepoints, which cannot be captured with a nonparametric test without splitting our data. (XLSX 10 kb)

Additional file 24: Supplementary zip file. Code and input tables used to generate the figures and tables in this paper. (ZIP 16846 kb)

## Abbreviations

AUC: Area under the curve
bp: Base pair
CARD: Comprehensive antibiotic resistance database
HMP: Human Microbiome Project
MDS: Multidimensional scaling
OTU: Operational taxonomic unit
PCoA: Principal coordinates analysis
ROC: Receiver operating characteristic
