# Supplementary figures and images for: Recent urbanization in China is correlated with a Westernized microbiome encoding increased virulence and antibiotic resistance genes
Source: Microbiome. 2017 Sep 15;5:121. doi: 10.1186/s40168-017-0338-7 (PMC5603068; doi:10.1186/s40168-017-0338-7)

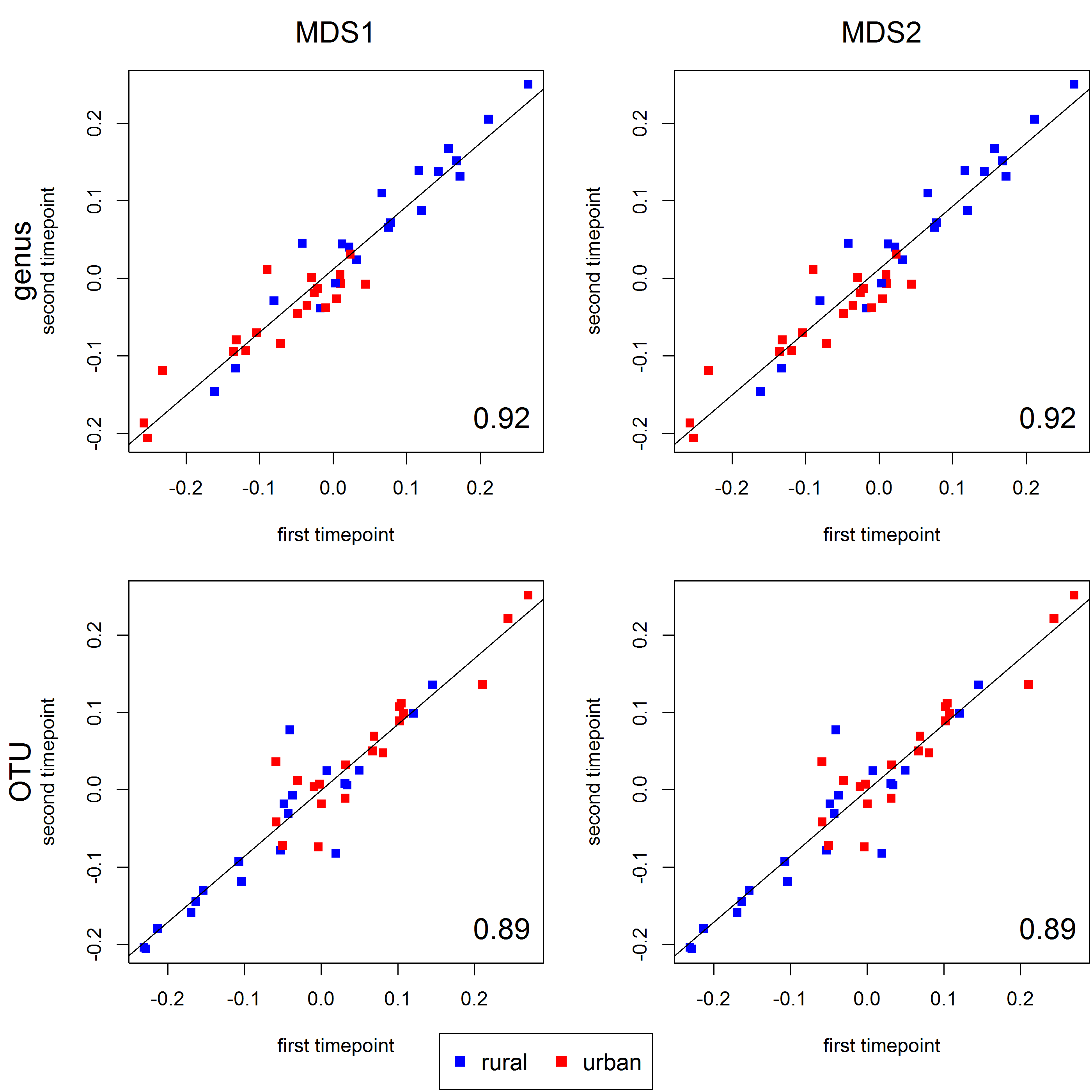

Supplement: Supplementary file 2 — Correlation between timepoints. Comparison between the first timepoint (x-axis) and second timepoint (y-axis) for the first (first column) and second (second column) axes of the PCoA plots for the 16S rRNA sequencing data at the genus and OTU taxonomic levels. Black line indicates the linear regression model and the number in the lower right indicates the multiple R 2 of that model. Urban samples are colored red while rural samples are blue. (TIFF 22968 kb) [file 40168_2017_338_MOESM2_ESM.tif]

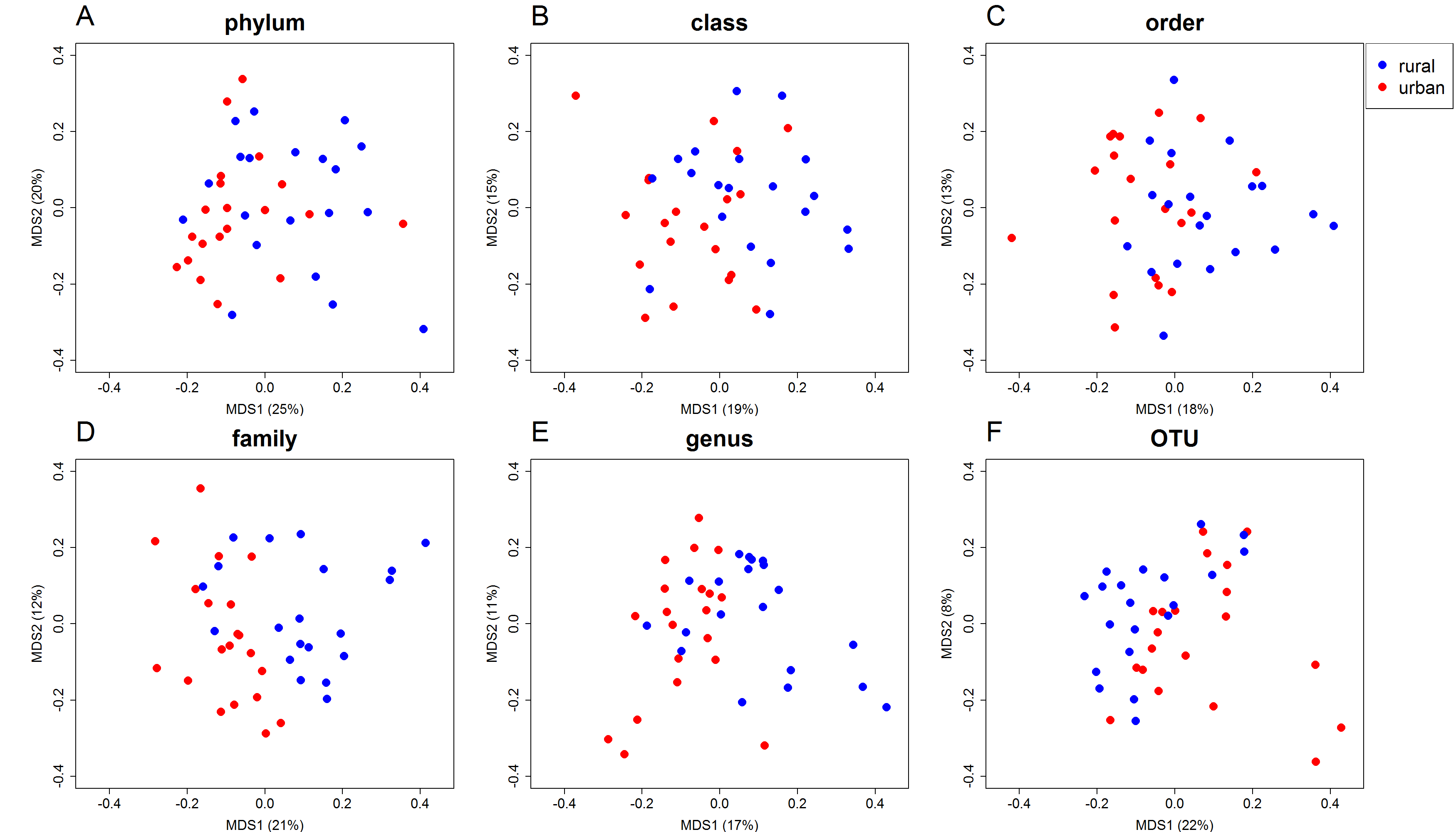

Supplement: Supplementary file 3 — Differences between urban and rural microbial composition is not dependent on bioinformatics pathway. (A–F) PCoA plot for each taxonomic level. Microbial composition was determined using the closed reference function of QIIME (as compared to RDP or Abundant OTU+ in Figs. 1 and 2) on the 16S rRNA sequencing data. Number in parentheses is the percent variation explained by that axis. (TIFF 20508 kb) [file 40168_2017_338_MOESM3_ESM.tif]

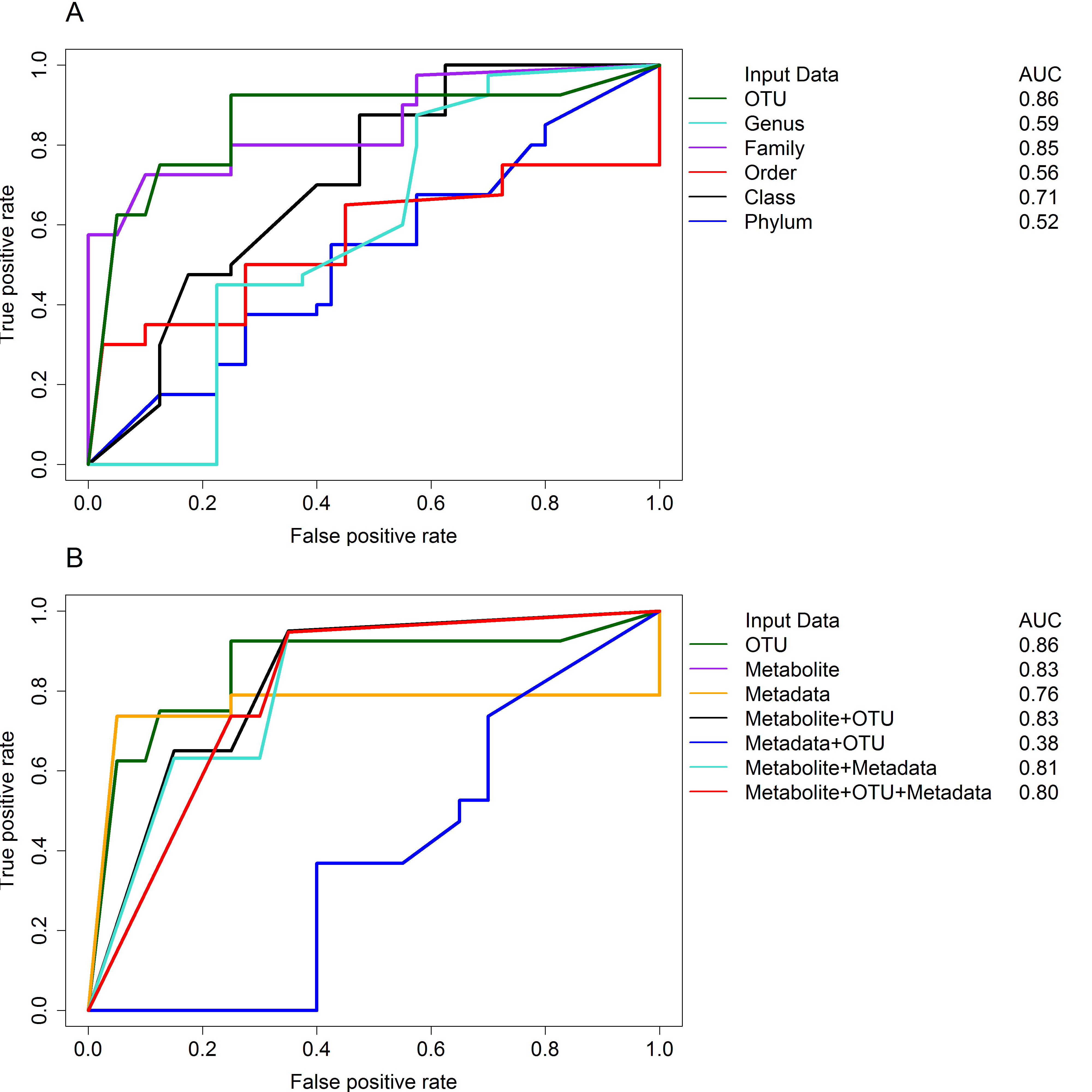

Supplement: Supplementary file 4 — Use of an alternative classifier confirms the ability to predict urban/rural status from the microbiome, the metabolome, or metadata. ROC curve generated from predictions from the rpart R function, using leave-one-out predictions of urban or rural status. The area under the curve (AUC) is indicated in the legend. (TIFF 46875 kb) [file 40168_2017_338_MOESM4_ESM.tif]

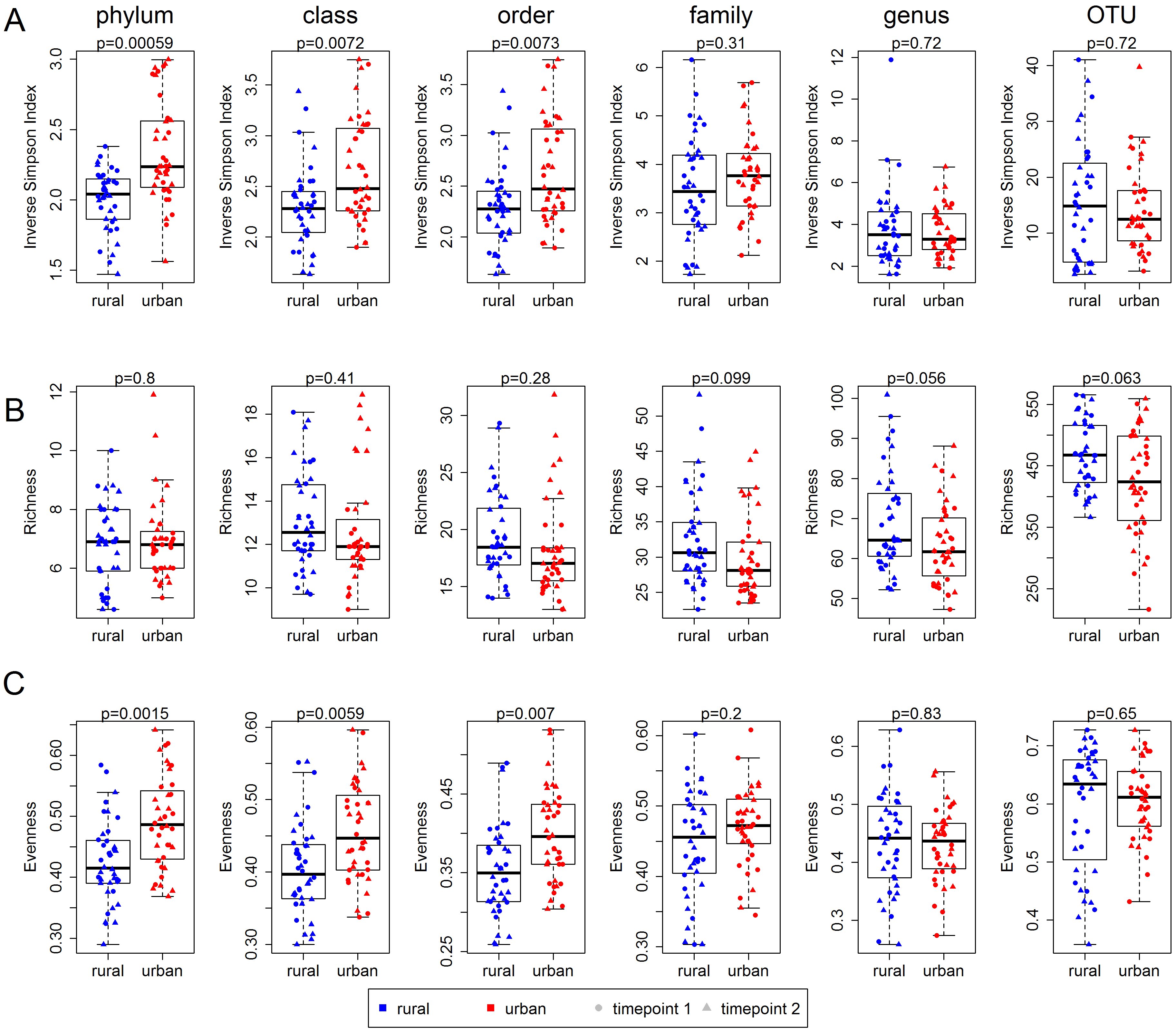

Supplement: Supplementary file 5 — Differences in microbial diversity based on 16S rRNA sequencing are driven by differences in evenness and are opposite differences in richness. Comparison of (A) inverse Simpson diversity index, (B) richness, or (C) evenness for each taxonomic level, based on 16S rRNA sequencing. P values indicate the significance of the difference between urban and rural subjects. Microbial composition was determined using RDP (phylum-genus) or AbundantOTU+ (OTU). (TIFF 34218 kb) [file 40168_2017_338_MOESM5_ESM.tif]

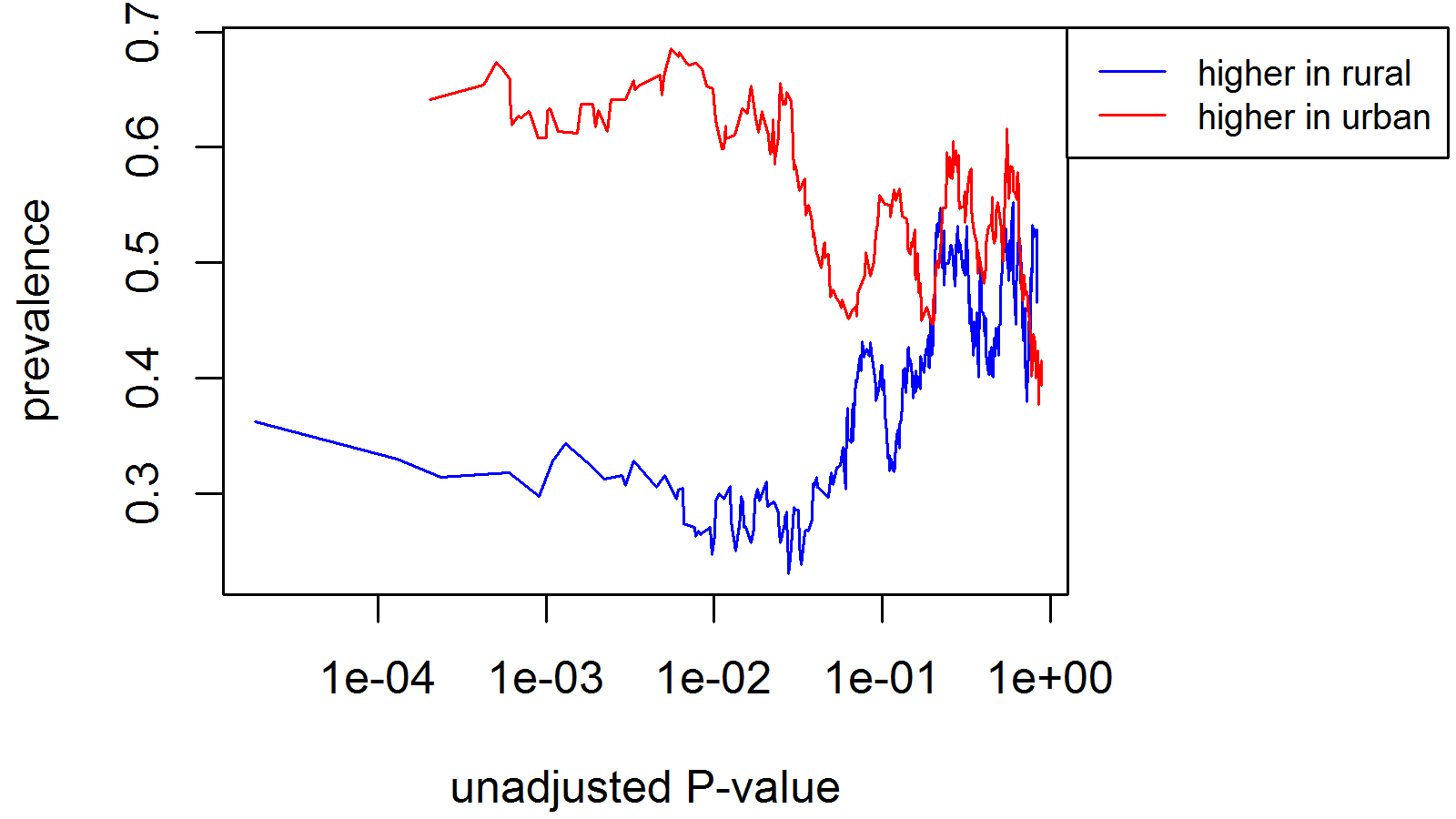

Supplement: Supplementary file 7 — Prevalence across the data set. Urban and rural prevalence vs. unadjusted P value for the null hypothesis that the OTU had the same distribution in rural and urban populations. Each point represents the average Human Microbiome Project prevalence of a 25 OTU windows. (TIFF 4218 kb) [file 40168_2017_338_MOESM7_ESM.tif]

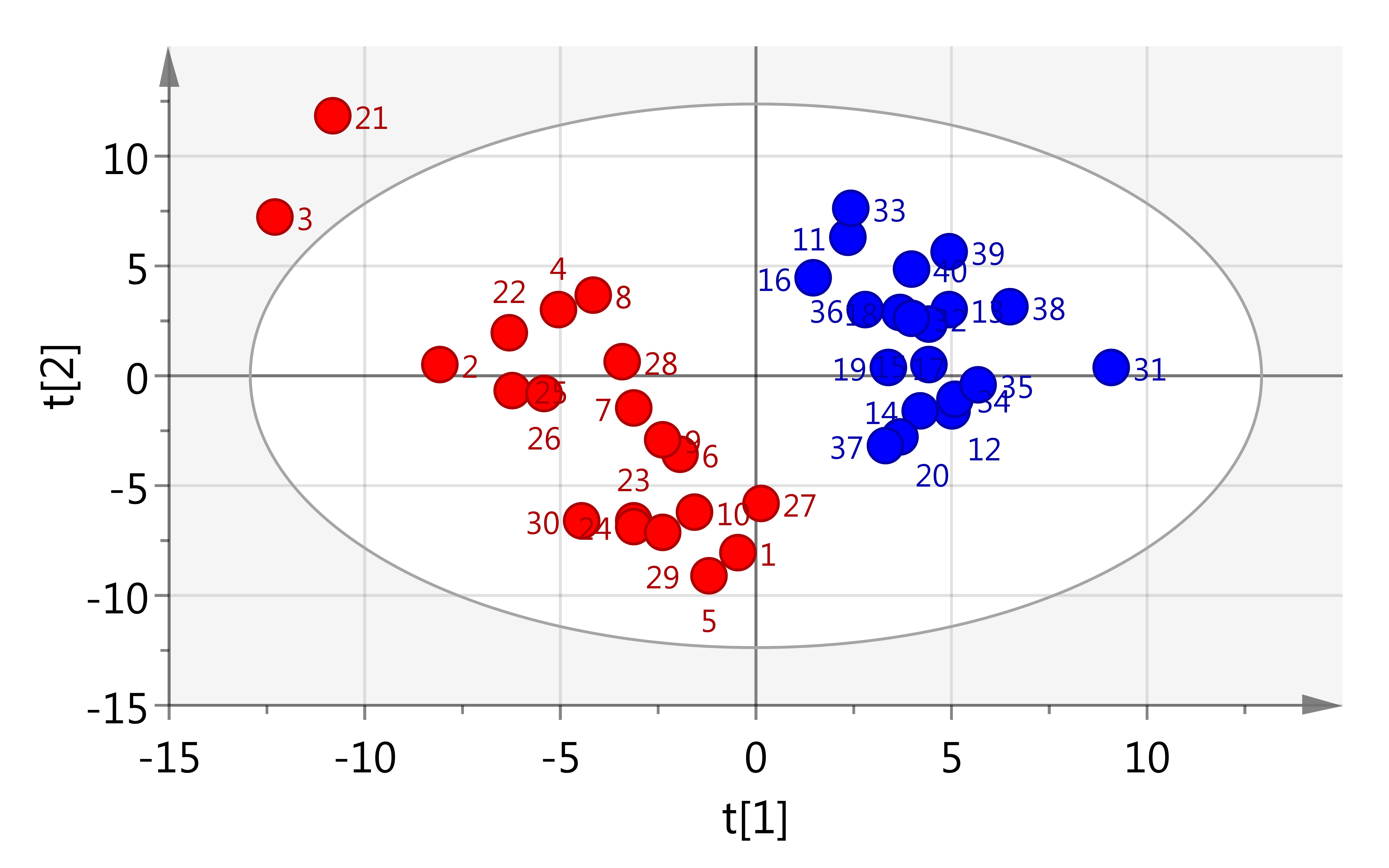

Supplement: Supplementary file 8 — PLS-DA confirms the separation of urban and rural samples using their metabolomic profiles. Score plot for the first two dimensions (t [1] and t [2]) defined by PLS-DA. Urban (red) and rural (blue) metabolomics profiles were very well separated by PLS-DA with R2Y = 0.963 (which indicates good model fit) and Q2Y = 0.637 (which indicates good predictability). Subject number is labeled on the plot. The separation passed permutation based validation. The predictability of this model was found to be better than any models built using the 999 permutated data sets. (JPEG 885 kb) [file 40168_2017_338_MOESM8_ESM.jpg]

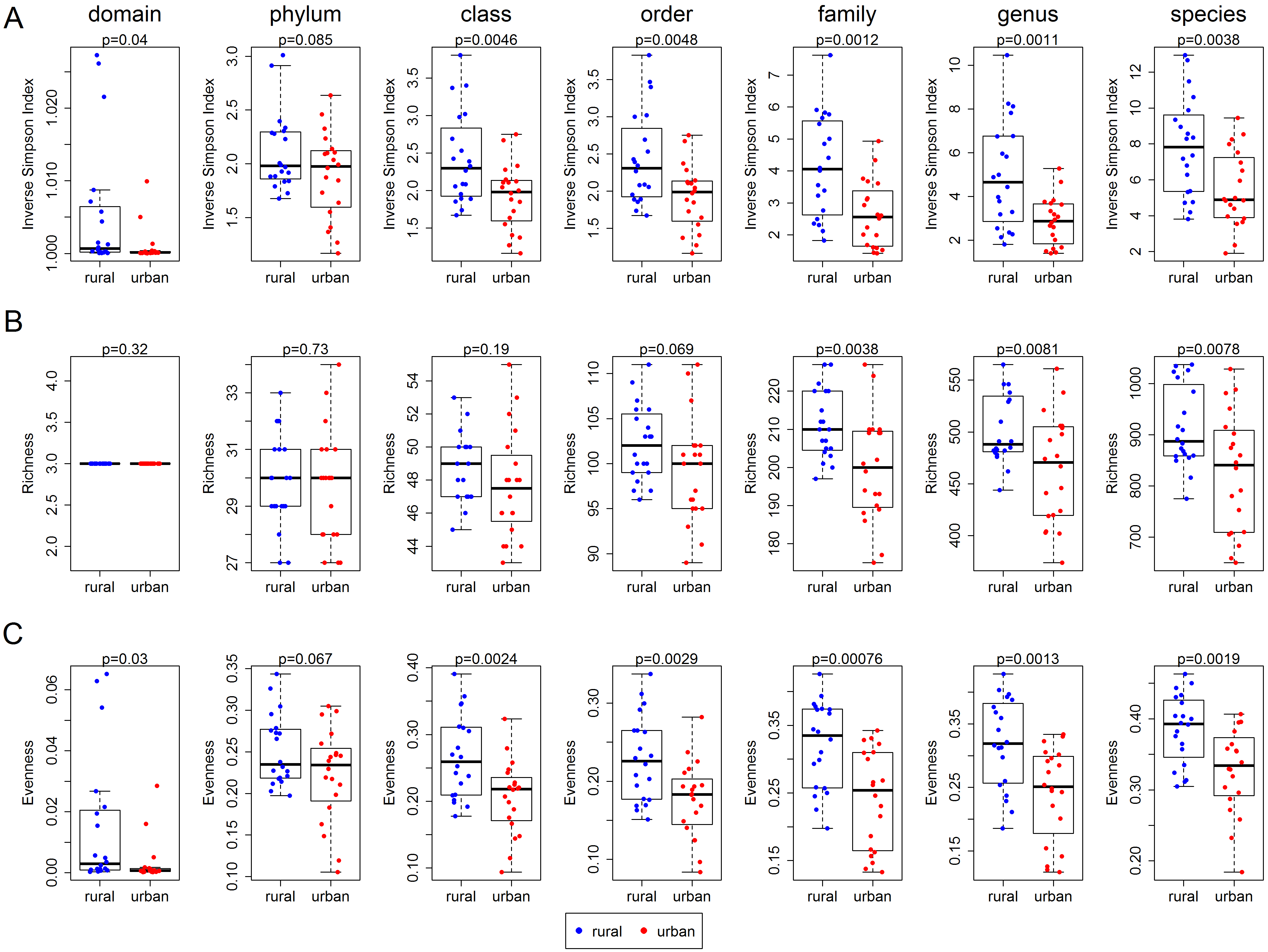

Supplement: Supplementary file 12 — Differences in microbial diversity based on whole genome sequencing is not dependent on diversity index used. Comparison of (A) inverse Simpson diversity index, (B) richness, or (C) evenness for each taxonomic level based on whole genome sequencing. P values indicate the significance of the difference between urban and rural subjects. (TIFF 39937 kb) [file 40168_2017_338_MOESM12_ESM.tif]

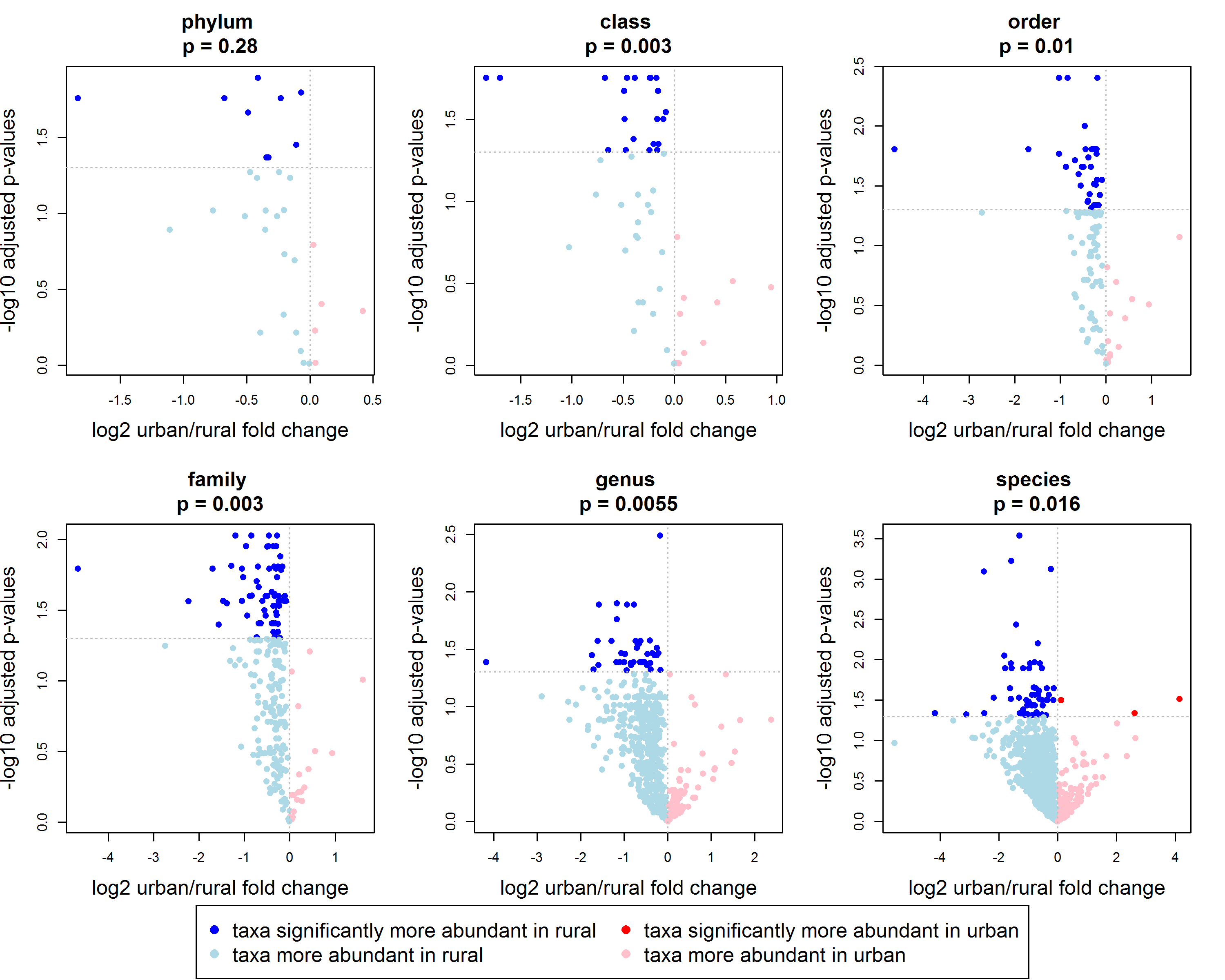

Supplement: Supplementary file 14 — Taxa significantly different in relative abundance between urban and rural subjects in whole genome sequencing have higher relative abundance in rural subjects. Volcano plots of the adjusted P values vs. fold change from whole genome sequencing at each taxonomic level. The P values given at the top of each plot were calculated using a chi-squared test. The horizontal dashed gray line indicates an adjusted P value of 0.05 while the vertical dashed gray line indicates an urban/rural fold change of 1. See Additional file 13: Table S5A-G for detailed model results for all taxa tested, including mean and standard deviation, P values, effect sizes (as measured by model R 2), and Spearman correlation. (TIFF 21093 kb) [file 40168_2017_338_MOESM14_ESM.tif]

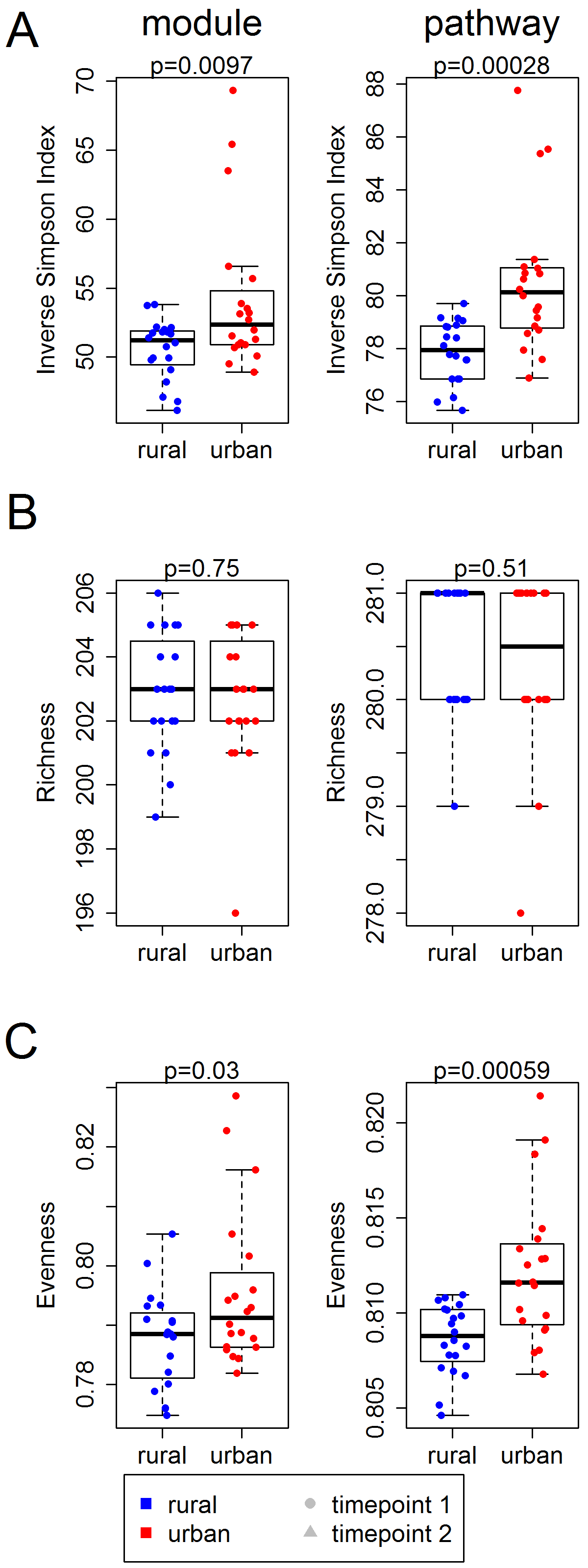

Supplement: Supplementary file 16 — Urban subjects have higher gene diversity and evenness but equal richness compared to rural subjects. Comparison of (A) inverse Simpson diversity index, (B) richness, or (C) evenness for KEGG modules (left column) and KEGG pathways (right column), based on whole genome sequencing. P values indicate the significance of the difference between urban and rural subjects. (TIFF 11250 kb) [file 40168_2017_338_MOESM16_ESM.tif]

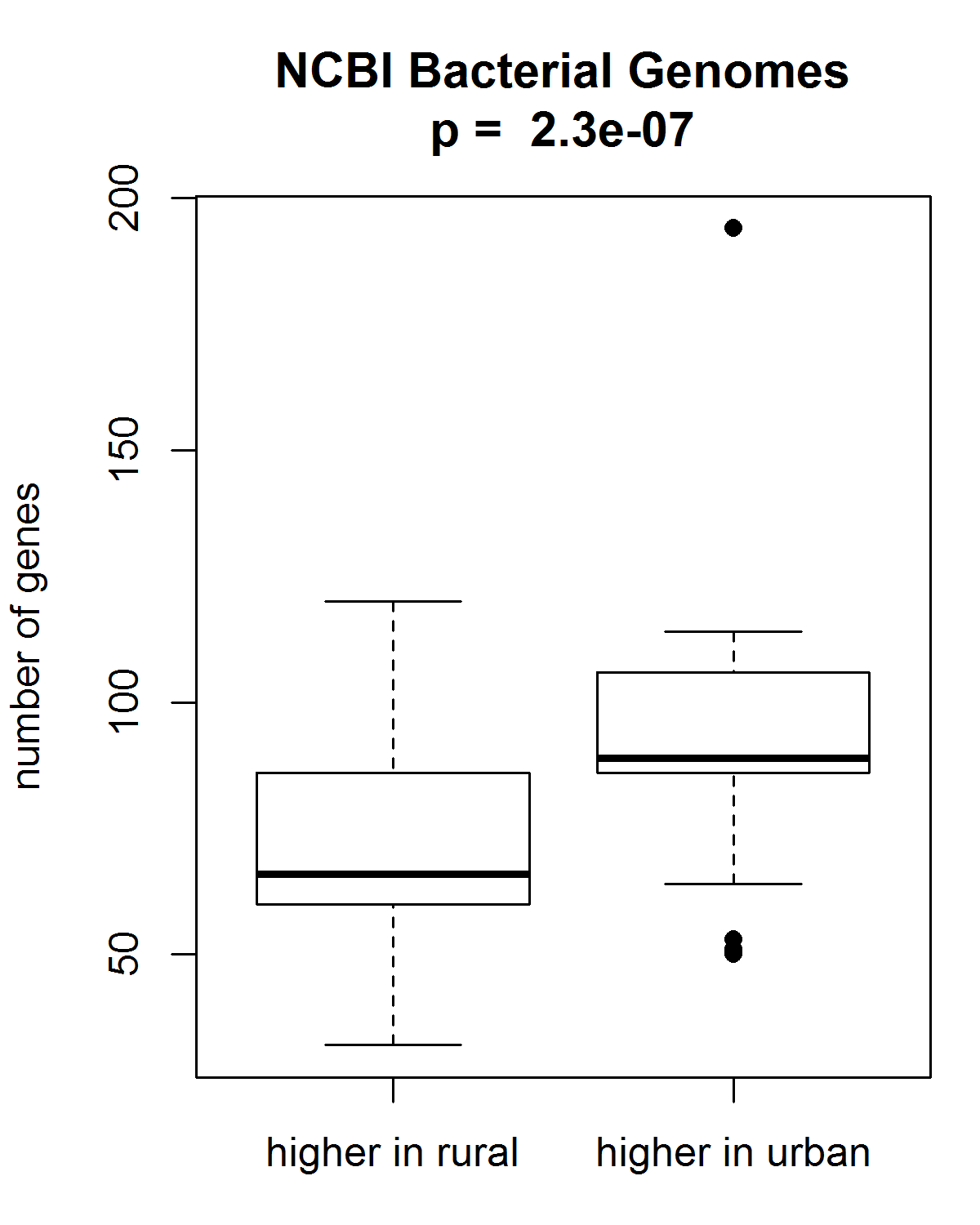

Supplement: Supplementary file 17 — Taxa more abundant in urban samples have more genes than taxa that are more abundant in rural samples. For each OTU, the number of annotated genes in the closest finished genome in NCBI (Fig. 2c) was counted. The P value is from a t test comparing the 80 OTU consensus sequences with higher relative abundance in rural samples to the 91 OTUs with higher relative abundance in urban samples. (TIFF 5273 kb) [file 40168_2017_338_MOESM17_ESM.tif]

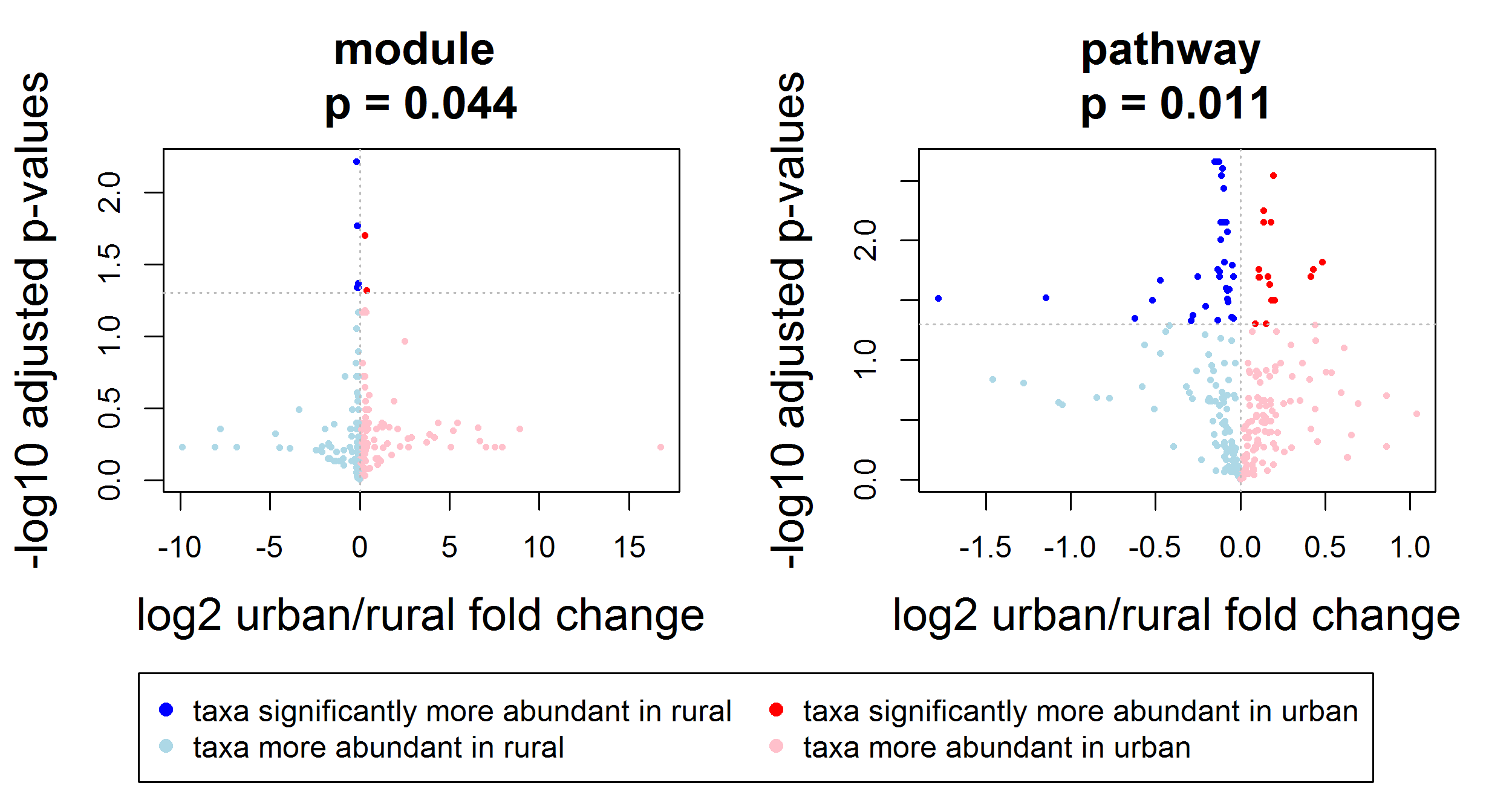

Supplement: Supplementary file 18 — KEGG pathways significantly different in relative abundance between urban and rural subjects tend to have a higher relative abundance in rural subjects. Volcano plots of the adjusted P values vs. fold change from whole genome sequencing for KEGG modules (left) and KEGG pathways (right). The P values given at the top of each plot were calculated using a chi-squared test. The horizontal dashed gray line indicates an adjusted P value of 0.05 while the vertical dashed gray line indicates an urban/rural fold change of 1. See Additional file 13: Table S5H-I for all KEGG functions tested and the model results, including mean and standard deviation, P values, effect sizes (as measured by model R 2), and Spearman correlation. (TIFF 9521 kb) [file 40168_2017_338_MOESM18_ESM.tif]

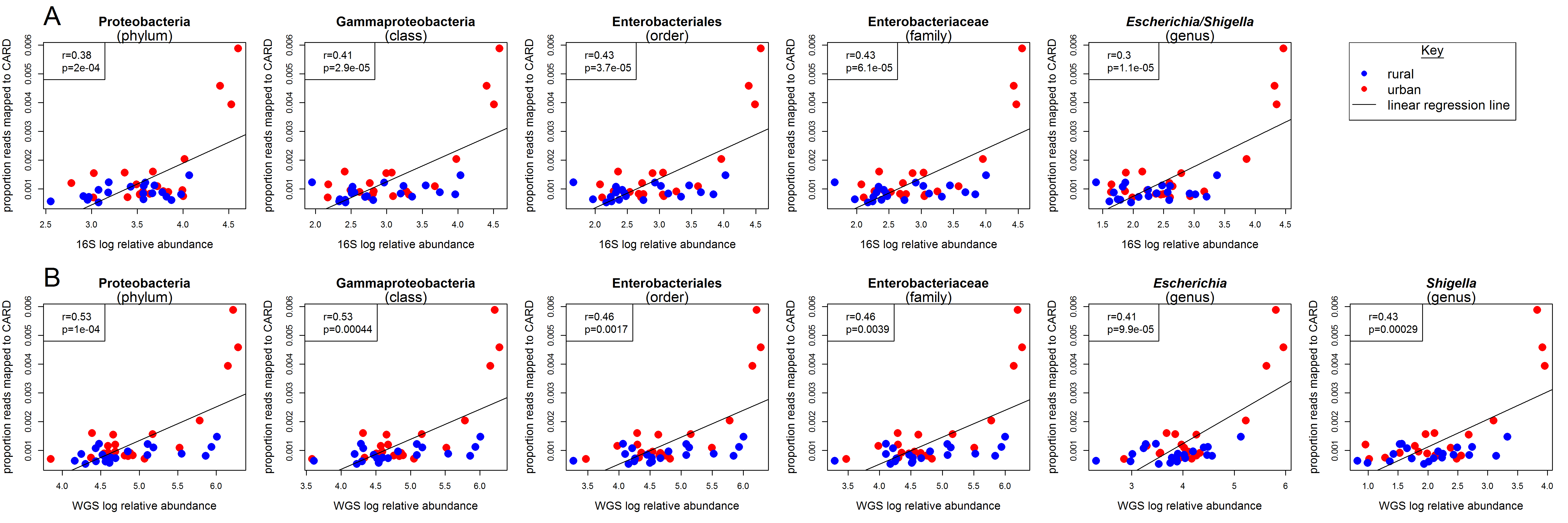

Supplement: Supplementary file 20 — The proportion of reads that map to genes that confer antibiotic resistance is associated with Escherichia and Shigella. Correlation between the proportion of whole genome sequencing reads that aligned to the CARD protein homolog database [31] (y-axis) to the relative abundance of the indicated taxa in that sample (x-axis). (A) The relative abundances were from 16S rRNA sequencing. (B) The relative abundances were from whole genome sequencing. The Escherichia and Shigella genera are shown, as well as each of the higher taxonomic levels that they belong to. All 14 OTUs classified into these genera were also significant (16S), as well as all six species identified in whole genome sequencing. In the upper left corner of each plot, r is the Pearson correlation coefficient and p is the Benjamini and Hochberg-adjusted P value from an ANOVA of the linear model. The model is indicated with the black line. See Additional file 19: Table S7 for further details. Similar patterns were seen with the proportion of whole genome sequencing reads that aligned to MvirDB (Additional file 21: Table S8). (TIFF 35156 kb) [file 40168_2017_338_MOESM20_ESM.tif]
