# Supplementary material for: Recent urbanization in China is correlated with a Westernized microbiome encoding increased virulence and antibiotic resistance genes
Source: Microbiome. 2017 Sep 15;5:121. doi: 10.1186/s40168-017-0338-7 (PMC5603068; doi:10.1186/s40168-017-0338-7)
Supplement: Supplementary file 22 — Supplementary Text. Detailed Methods. (DOCX 324 kb) [file 40168_2017_338_MOESM22_ESM.docx]

# Methods

## Study Participants

We used a subset of individuals (n=40) from a larger study on arsenic exposure (n=150) in Hunan Province, located in Southern China. Two communities that differed by degree of urbanization were selected for the microbiome subsample (Table 1). Both communities are in the subtropical maritime monsoon climate zone, with similar regional diet behaviors that differed by degree of Westernization of the diet. A random sample of individuals aged 18-65 were selected for the study, with exclusion of individuals who lived in these communities for less than two years, were pregnant or lactating, had known cancer, worked in an occupation with potential exposure to arsenic (due to the focus of the larger study on arsenic), or had antibiotic use within the past three months.

Data were collected across a three-day house visit by trained China CDC data collectors, who completed a three-day dietary recall and collected anthropometric, blood pressure, and sociodemographic data as well as fasting blood, spot urine and fecal samples. Participants were given the QIAGEN collection and extraction kit (QIAGEN, Hilden, Germany) at the start of the three-day visit using a modification of the Human Microbiome Project (HMP) [1] protocol and samples were collected as completed. The HMP protocol was modified for Chinese language and for use with Fisherbrand commode specimen collection system. Participants were provided a small cooler and ice pack to store in freezer until ready for use. Interviewers were telephoned immediately after fecal collection and samples stored in freezer for pickup within 24 hours of collection and then placed into -80 C for storage and cold-chain shipping to BGI for sequencing.

Whole blood was collected and immediately centrifuged and stored at -80 degrees until metabolomic profiling of the serum and biomarker analysis, with the exception of glucose, which was immediately assayed. Spot urine was collected and assayed for inorganic arsenic measures. In addition, we conducted a repeat fecal collection in all 40 participants to characterize stability in gut microbiota over a 2 week period.

## Metabolite Analysis

Fasting blood was collected and immediately refrigerated and within 48 hours centrifuged to separate plasma, which was stored at -80 degrees and cold-chain shipped to Metabolon, China. Metabolites were assayed using Chromatography/Mass Spectrometry on the Metabolon Platform (Metabolon, Inc.) in an untargeted approach validated with quantitative assays against calibration standards and stable isotopic-labeled analyses [2]. Bioinformatics included data extraction into a relational database and peak identification software; proprietary data processing tools for QC and compound identification; and a collection of interpretation and visualization tools for use by data analysts. Metabolon cross-matches mass-spectra data obtained in a study against the Metabolon library of standards identified by a proprietary ion clustering algorithm, by matching purified standard or by classical structural analysis.

## Metadata

Height was measured without shoes to the nearest 0.2 cm using a portable Seca stadiometer, and weight was measured without shoes and in light clothing to the nearest 0.1 kg on a calibrated floor scale, to generate BMI (kg/m^2^). Waist circumference was measured midway between the lowest rib and the iliac crest using a nonelastic tape. Diet data were collected using three consecutive 24-hour recalls [3] **at the individual level and a food inventory at the household level occurring during the same 3-day period to collect diet data, randomly starting from Monday to Sunday. For 24-hour recalls, trained interviewers recorded types and amounts of food, types of meal, and places of food preparation of all food items consumed by each household member. All foods available in the household were measured daily for the food inventory. We used a Chinese food composition table to estimate nutrient intake of reported foods [4]. This dietary assessment** has been validated relative to doubly labeled water (Pearson correlation coefficient men: 0.56; women: 0.60) for energy [5] and urine for sodium (Pearson correlation coefficient: 0.58), and potassium (Pearson correlation coefficient: 0.59) [6]. Daily dietary intake was calculated as the average across all three days. Self-reported questionnaires were used to collect information regarding gender, age, probiotic use, and antibiotic use in the past 6 months. The CNHS collected blood samples by venipuncture after an overnight fast. Biomarkers (including glucose, HbA1c, insulin, CRP, HDL, LDL, triglycerides and total cholesterol) as well as urinary inorganic arsenic exposure were measured with standard procedures. Morning and spot urine samples were collected to measure urinary inorganic arsenic exposure; these two measures were averaged and log transformed due to highly skewed distribution.

## 16S rRNA Sequencing

Paired-end 16S rRNA sequencing targeting the V4 hypervariable region was performed by Beijing Genomics Institute (BGI)-Shenzhen, with an average of 106,812 reads per sample (Table S1), using an Illumina MiSeq PE250. PCR amplification was performed on 30ng of DNA using the NEB Phusion High-Fidelity PCR Master Mix, with the 515F (GTGCCAGCMGCCGCGGTAA) and 806R (GGACTACHVGGGTWTCTAAT) primers. Our PCR program was 98 ^o^C for 3 minutes, then 30 cycles of 98 ^o^C for 45 seconds, 55 ^o^C for 45 seconds, and 72 ^o^C for 45 seconds, with a final extension of 72 ^o^C for 7 minutes. PCR products were purified with AMPure XP beads (Agencourt) and quantified using an Agilent 2100 Bioanalzyer with Agilent DNA 1000 Reagents, then sequenced on an Illumina MiSeq PE250, following manufacturer’s instruction.

Paired reads were merged if they overlapped by at least 200 bases with at least 95% similarity, using QIIME’s join_paired_ends.py (version 1.8.0) [7]. The resulting sequences were cleansed to contain a minimum Phred quality score of 20 over 95% of the read length. The forward reads were clustered using AbundantOTU+ (v0.93b) [8] with the “-abundantonly” option at 97% similarity. Chimeras were removed using uchime (v.4.2.40) [9] and the Gold reference database. Taxonomic ranks were also assigned for the merged reads through QIIME (Quantitative Insights Into Microbial Ecology) version 1.8.0 [7] or for the forward reads using the RDP classifier version 2.2 [10] with confidence set to 80%. OTU consensus sequences were then aligned to GreenGenes using PyNAST [11]. Raw OTU counts were normalized and log_10_ transformed [12] using the following formula:

$${log}_{10}\left( \frac{RC}{n} x \frac{\sum x}{N}+1 \right)$$

where RC is the read count for a particular taxa in a particular sample, *n* is the total number of reads in that sample, the sum of *x* is the total number of reads in all samples and N is the total number of samples. In addition, raw counts were rarified using a subsample size equal to the sample with the fewest reads at that taxonomic level, using the rrarefy function of vegan [13]. The Shannon diversity index and inverse Simpson index were then calculated using the diversity function of vegan [13], while richness was calculated using the specnumber function, and evenness was calculated as the Shannon Index divided by the natural log of the richness. This was repeated 10 times, and the average value was used.

In order to compare taxa in our study to other databases (Fig. 2B-E), the consensus sequences from the AbundantOTU+ pipeline were used as queries against the Silva database (SILVA_123_SSURef_tax_silva.fasta; http://www.arb-silva.de/download/archive/), the NCBI collection of assembled microbial genomes (all.frn.tar.gz as downloaded from ftp://ftp.ncbi.nih.gov/genomes/Bacteria on October 15, 2015) and the HMP "most wanted" clusters (http://hmpdacc.org/most_wanted/#data). Mapping each query consensus sequence to each reference database was accomplished using best BLAST hits (blastn with default parameters) as resulting from ncbi-blast-2.2.29+ [14].

## Whole Genome Sequencing

Whole genome sequencing was performed by BGI-Shenzhen, which generated 10 million reads per sample using an Illumina HiSeq 4000 with a 150-180 base pair (bp) short insert library and 101bp paired end sequencing (Table S1), using the manufacturer’s instruction (Illumina), with the HiSeq 4000 PE Cluster Kit (Illumina) and HiSeq 4000 SBS Kit (Illumina )[15].

Reads were filtered to remove any reads that met any of the following requirements: (1) the read contained >5 Ns (2) >40% of the bases had a quality <20 (3) >44% of the adapter was present in the read. The reads were aligned to the Genome Reference Consortium Human Build 38, which was downloaded from the UCSC Genome Browser (http://hgdownload.cse.ucsc.edu/downloads.html#human). Any read that mapped to this human reference genome was filtered.

Microbial composition was determined using Kraken [16], using the default settings and the MiniKraken DB. Counts were normalized using the same transformation we used for the 16S rRNA data. KEGG module and pathway abundance was determined by using tblastx (using ncbi-blast-2.2.29+) [14] with default parameters to align the reads to the KEGG database [17] and then using HUMAnN [18] to generate abundance tables. No transformation of this output was performed. Diversity measures were calculated the same as for the 16S rRNA sequencing, except no rarefaction was performed since the samples were already rarefied to 10 million reads prior to analysis.

## Antibiotic Resistance and Virulence Factors

To assess the presence of genes that confer antibiotic resistance, all whole genome sequencing reads were aligned to the Comprehensive Antibiotic Resistance Database (CARD) protein homolog database, version 1.0.4 [19] using the BWA (Burrows-Wheeler Alignment) [20]. Similarly, to assess the presence of virulence factors, BWA was used to align the whole genome sequencing reads to MvirDB (downloaded September 16, 2016) [21]. Any read that mapped anywhere in the database was counted as a hit, and the number of reads that aligned in each sample was divided by 10 million (the number of reads) to get the proportion of reads that aligned to the CARD or MvirDB database.

## Statistical Analysis

The Principal Coordinate Analysis (PCoA) was generated from the Bray-Curtis distance of the normalized and log_10_ transformed microbiome counts, HUMAnN output or the scaled counts from Metabolon using the capscale function in the vegan R package [13]. Classification in the main text was performed by running the cforest function in the R package party [22] on the dataset, using leave-one-out predictions. The results were confirmed using the rpart function in the rpart R package [23]. Receiver Operating Characteristic (ROC) curves were generated with the ROCR package [24].

For the 16S rRNA microbiome data, we used a generalized mixed linear model of the form:

abundance ~ timepoint + ruralUrban + 1|subject + ɛ

where abundance is the log_10_ normalized abundance of a particular OTU, timepoint indicates the timepoint the sample was taken (timepoint 1 or 2), ruralUrban indicates whether the subject lives in a rural or urban setting, and 1|subject indicates that we used the subject ID as a random effect. We filtered any taxa absent in more than one quarter of the samples. The model was fit using the lme function in the R nlme package, with the REML method [25, 26]. We then ran an ANOVA analysis on the above model to generate P-values for time and rural/urban status. The P-values for subject reported in the Table S2 were calculated using an ANOVA of this model and a model with the subject ID removed (abundance ~ timepoint + ruralUrban + ɛ). Model R^2^ and Spearman correlations can also be found in Table S2. Individual plots for each taxon are provided at https://github.com/kwinglee/UrbanRuralChina/tree/master/individualPlotsForAllData. In addition, we used the Wilcoxon rank sum test on each timepoint individually to confirm the linear model urban vs. rural results, using a nonparametric test that has fewer assumptions than a mixed linear model. The P-values were then adjusted for multiple hypothesis testing using the method of Benjamini & Hochberg [27]. These same models were used to calculate P-values for the diversity measures and PCoA axes, replacing abundance with the diversity measure or PCoA orthonormal site scores.

For the whole genome sequencing data, metadata and metabolome data, we used a model of the form:

abundance ~ ruralUrban + ɛ

where abundance is the microbiome taxa or gene relative abundance, metabolite abundance or metadata value for each measured value. For the whole genome sequencing data, we filtered any taxa or gene absent in more than one quarter of the samples. These models were fit using the using the R function lm [26]. We then ran an ANOVA analysis on the above models to generate P-values. Model R^2^ and Spearman correlations can also be found in the supplemental tables. Individual plots for each taxon and gene from whole genome sequencing, as well as for each metabolite and metadata are provided at https://github.com/kwinglee/UrbanRuralChina/tree/master/individualPlotsForAllData. In order to further validate our findings, we also ran a Wilcoxon rank sum test on rural vs. urban status for the whole genome sequencing data, metadata and metabolome data. These values were highly correlated with the linear model results (Methods Figure M1 and Supplemental Table S9). For categorical metadata (gender, probiotic use and antibiotic use), we used a Fisher exact test. P-values were adjusted for multiple hypothesis testing using the method of Benjamini & Hochberg [27]. Similar models were run for the PCoA orthonormal site scores to generate the P-values for each axis and on the diversity measures to generate the diversity P-values. P-values on the volcano plots in Figs. S9 and S11 were calculated using a chi-square test.

For the metabolome, metadata and WGS results, which only had one timepoint, we compared the number of significant results from the linear models to the number of significant results from the nonparametric models, which are less sensitive to normalization schemes, outliers, and make fewer assumptions about the normality of the data. As Methods Figure M1 and Supplemental Table S9 shows, the p-values were highly correlated between the models and the majority of the significant results were statistically significant with both linear models and nonparametric (Wilcoxon) models. Thus, we utilized the results from linear models so that we could account for the fact that in our 16S rRNA sequencing data we had multiple samples from the same individual at two timepoints, which we cannot do with a nonparametric test without splitting our data.

To identify significant associations between the microbiome, metabolome, metadata, antibiotic resistance genes or virulence factors, we used a simple linear model of the form:

*y* = *mx* + *b* + ɛ

where *m* is the slope, b the intercept, *x* the abundance for one of the measured values (microbiome abundance, metabolite, metadata, or proportion of reads mapped) and *y* the abundance for the other value being compared. Any taxa or genes from the sequencing data that were not detected in more than a quarter of the samples were filtered from these associations. These models were fit using the R function lm [26]. We then ran an ANOVA analysis on the above model to generate P-values. Model R^2^ and Spearman correlations can also be found in the supplemental tables. In order to further validate our findings, we calculated the Kendall rank correlation between the two measured values, unless one value was a categorical metadata, in which case we used a Wilcoxon rank sum. P-values were then adjusted for multiple hypothesis testing using the method of Benjamini & Hochberg [27].

In addition, Partial Least Square Discriminant Analysis (PLS-DA) in SIMCA (Version 14, Umetrics, Umeå, Sweden) was used to detect metabolites that can best distinguish the two groups (urban vs. rural). The default 7-round cross-validation in SIMCA was used to compute the diagnostic Q^2^Y value. Permutation based validation was used to prevent overfitting, using 999 permuted data sets. A model was considered valid if its prediction ability was found to be better than 95% of the models built using the permutated data sets.

# Supplemental Methods Figure


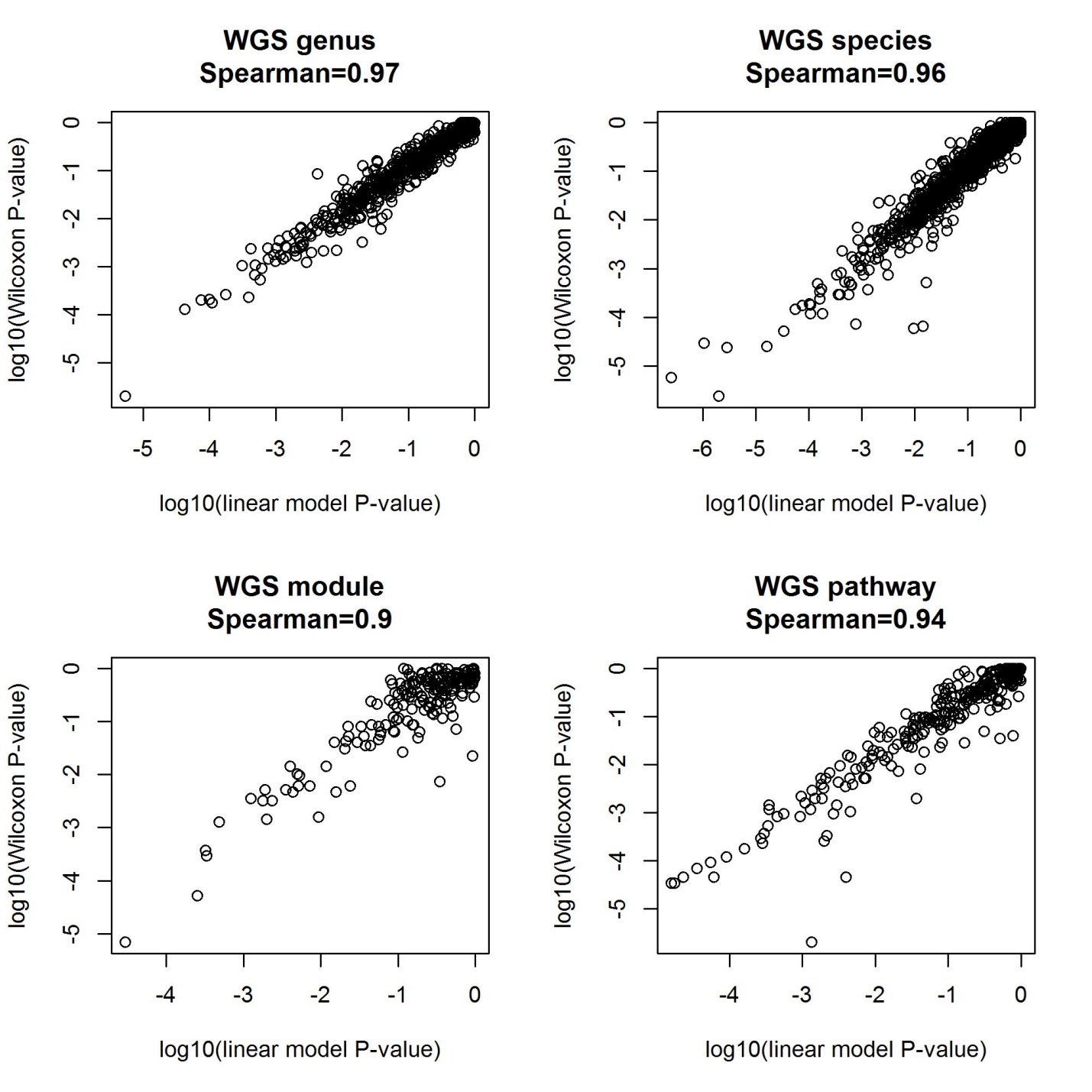


**Supplemental Methods Figure M1. Linear model and nonparametric P-values are highly correlated.**

Comparison of the unadjusted P-values from a Wilcoxon rank sum test to the linear model unadjusted P-values for the whole genome sequencing (WGS) data. The Spearman correlation is given in the title.

# References

1. Aagaard K, Petrosino J, Keitel W, Watson M, Katancik J, Garcia N, Patel S, Cutting M, Madden T, Hamilton H, Harris E, Gevers D, Simone G, McInnes P, Versalovic J. The Human Microbiome Project strategy for comprehensive sampling of the human microbiome and why it matters. FASEB J. 2013;27:1012-1022.

2. Evans AM, DeHaven CD, Barrett T, Mitchell M, Milgram E. Integrated, nontargeted ultrahigh performance liquid chromatography/electrospray ionization tandem mass spectrometry platform for the identification and relative quantification of the small-molecule complement of biological systems. Anal Chem. 2009;81:6656-6667.

3. Du S, Mroz TA, Zhai F, Popkin BM. Rapid income growth adversely affects diet quality in China--particularly for the poor! Soc Sci Med. 2004;59:1505-1515.

4. Yang Y. Chinese food composition table 2004**.** Beijing: Peking University Medical Press. 2005.

5. Yao M, McCrory MA, Ma G, Tucker KL, Gao S, Fuss P, Roberts SB. Relative influence of diet and physical activity on body composition in urban Chinese adults. Am J Clin Nutr. 2003;77:1409-1416.

6. Du S, Batis C, Wang H, Zhang B, Zhang J, Popkin BM. Understanding the patterns and trends of sodium intake, potassium intake, and sodium to potassium ratio and their effect on hypertension in China. Am J Clin Nutr. 2014;99:334-343.

7. Caporaso JG, Kuczynski J, Stombaugh J, Bittinger K, Bushman FD, Costello EK, Fierer N, Pena AG, Goodrich JK, Gordon JI, Huttley GA, Kelley ST, Knights D, Koenig JE, Ley RE, Lozupone CA, McDonald D, Muegge BD, Pirrung M, Reeder J, Sevinsky JR, Turnbaugh PJ, Walters WA, Widmann J, Yatsunenko T, Zaneveld J, Knight R. QIIME allows analysis of high-throughput community sequencing data. Nat Methods. 2010;7:335-336.

8. Ye Y. Identification and Quantification of Abundant Species from Pyrosequences of 16S rRNA by Consensus Alignment. Proceedings (IEEE Int Conf Bioinformatics Biomed). 2011;2010:153-157.

9. Edgar RC, Haas BJ, Clemente JC, Quince C, Knight R. UCHIME improves sensitivity and speed of chimera detection. Bioinformatics. 2011;27:2194-2200.

10. Wang Q, Garrity GM, Tiedje JM, Cole JR. Naive Bayesian classifier for rapid assignment of rRNA sequences into the new bacterial taxonomy. Appl Environ Microbiol. 2007;73:5261-5267.

11. Caporaso JG, Bittinger K, Bushman FD, DeSantis TZ, Andersen GL, Knight R. PyNAST: a flexible tool for aligning sequences to a template alignment. Bioinformatics. 2010;26:266-267.

12. McCafferty J, Muhlbauer M, Gharaibeh RZ, Arthur JC, Perez-Chanona E, Sha W, Jobin C, Fodor AA. Stochastic changes over time and not founder effects drive cage effects in microbial community assembly in a mouse model. ISME J. 2013;7:2116-2125.

13. Oksanen JB, F. Guillaume; Kindt, Roeland; Legendre, Pierre; Minchin, Peter R. ; O'Hara, R. B. ; Simpson, Gavin L. ; Solymos, Peter ; Stevens, M. Henry H. ; Wagner, Helene vegan: Community Ecology Package. <http://CRAN.R-project.org/package=vegan>. Accessed 8 Oct 2015.

14. Altschul SF, Gish W, Miller W, Myers EW, Lipman DJ. Basic local alignment search tool. J Mol Biol. 1990;215:403-410.

15. Qin J, Li Y, Cai Z, Li S, Zhu J, Zhang F, Liang S, Zhang W, Guan Y, Shen D, Peng Y, Zhang D, Jie Z, Wu W, Qin Y, Xue W, Li J, Han L, Lu D, Wu P, Dai Y, Sun X, Li Z, Tang A, Zhong S, Li X, Chen W, Xu R, Wang M, Feng Q, Gong M, Yu J, Zhang Y, Zhang M, Hansen T, Sanchez G, Raes J, Falony G, Okuda S, Almeida M, LeChatelier E, Renault P, Pons N, Batto JM, Zhang Z, Chen H, Yang R, Zheng W, Li S, Yang H, Wang J, Ehrlich SD, Nielsen R, Pedersen O, Kristiansen K, Wang J. A metagenome-wide association study of gut microbiota in type 2 diabetes. Nature. 2012;490:55-60.

16. Wood DE, Salzberg SL. Kraken: ultrafast metagenomic sequence classification using exact alignments. Genome Biol. 2014;15:R46.

17. Kanehisa M, Goto S. KEGG: kyoto encyclopedia of genes and genomes. Nucleic Acids Res. 2000;28:27-30.

18. Abubucker S, Segata N, Goll J, Schubert AM, Izard J, Cantarel BL, Rodriguez-Mueller B, Zucker J, Thiagarajan M, Henrissat B, White O, Kelley ST, Methe B, Schloss PD, Gevers D, Mitreva M, Huttenhower C. Metabolic reconstruction for metagenomic data and its application to the human microbiome. PLoS Comput Biol. 2012;8:e1002358.

19. McArthur AG, Waglechner N, Nizam F, Yan A, Azad MA, Baylay AJ, Bhullar K, Canova MJ, De Pascale G, Ejim L, Kalan L, King AM, Koteva K, Morar M, Mulvey MR, O'Brien JS, Pawlowski AC, Piddock LJ, Spanogiannopoulos P, Sutherland AD, Tang I, Taylor PL, Thaker M, Wang W, Yan M, Yu T, Wright GD. The comprehensive antibiotic resistance database. Antimicrob Agents Chemother. 2013;57:3348-3357.

20. Li H, Durbin R. Fast and accurate short read alignment with Burrows-Wheeler transform. Bioinformatics. 2009;25:1754-1760.

21. Zhou CE, Smith J, Lam M, Zemla A, Dyer MD, Slezak T. MvirDB--a microbial database of protein toxins, virulence factors and antibiotic resistance genes for bio-defence applications. Nucleic Acids Res. 2007;35:D391-394.

22. Strobl C, Boulesteix AL, Kneib T, Augustin T, Zeileis A. Conditional variable importance for random forests. BMC Bioinformatics. 2008;9:307.

23. Therneau TA, Beth; Ripley, Brian rpart: Recursive Partitioning and Regression Trees. <http://CRAN.R-project.org/package=rpart>. Accessed 9 Oct 2015.

24. Sing T, Sander O, Beerenwinkel N, Lengauer T. ROCR: visualizing classifier performance in R. Bioinformatics. 2005;21:3940-3941.

25. Pinheiro J, Bates D, DebRoy S, Sarkar D, R Core Team. nlme: Linear and Nonlinear Mixed Effects Models. <http://CRAN.R-project.org/package=nlme>. Accessed 27 Jan 2016.

26. R Core Team. R: A Language and Environment for Statistical Computing. <http://www.R-project.org/>. Accessed 27 Jan 2016.

27. Benjamini Y, Hochberg Y. Controlling the false discovery rate: a practical and powerful approach to multiple testing. J Roy Statist Soc Ser B (Methodological). 1995;57.
